# Transcriptome- and proteome-wide effects of a circular RNA encompassing four early exons of the spinal muscular atrophy genes

**DOI:** 10.1038/s41598-024-60593-7

**Published:** 2024-05-07

**Authors:** Diou Luo, Eric W. Ottesen, Ji Heon Lee, Ravindra N. Singh

**Affiliations:** Department of Biomedical Sciences, College of Veterinary Medicine, Iowa State University, Ames, IA 50011 USA

**Keywords:** Spinal muscular atrophy, SMA, Survival Motor Neuron, SMN, Circular RNA, circRNA, Proteome, Transcriptome, Alternative splicing, Proteomics, Transcriptomics

## Abstract

Spinal muscular atrophy (SMA) genes, *SMN1* and *SMN2* (hereinafter referred to as *SMN1/2*), produce multiple circular RNAs (circRNAs), including C2A–2B–3–4 that encompasses early exons 2A, 2B, 3 and 4. C2A-2B-3-4 is a universally and abundantly expressed circRNA of *SMN1/2*. Here we report the transcriptome- and proteome-wide effects of overexpression of C2A–2B–3–4 in inducible HEK293 cells. Our RNA-Seq analysis revealed altered expression of ~ 15% genes (4172 genes) by C2A–2B–3–4. About half of the affected genes by C2A–2B–3–4 remained unaffected by L2A–2B–3–4, a linear transcript encompassing exons 2A, 2B, 3 and 4 of *SMN1*/*2*. These findings underscore the unique role of the structural context of C2A–2B–3–4 in gene regulation. A surprisingly high number of upregulated genes by C2A–2B–3–4 were located on chromosomes 4 and 7, whereas many of the downregulated genes were located on chromosomes 10 and X. Supporting a cross-regulation of *SMN1*/*2* transcripts, C2A–2B–3–4 and L2A–2B–3–4 upregulated and downregulated *SMN1*/*2* mRNAs, respectively. Proteome analysis revealed 61 upregulated and 57 downregulated proteins by C2A–2B–3–4 with very limited overlap with those affected by L2A–2B–3–4. Independent validations confirmed the effect of C2A–2B–3–4 on expression of genes associated with chromatin remodeling, transcription, spliceosome function, ribosome biogenesis, lipid metabolism, cytoskeletal formation, cell proliferation and neuromuscular junction formation. Our findings reveal a broad role of C2A–2B–3–4, and expands our understanding of functions of *SMN1/2* genes.

## Introduction

Circular RNAs (circRNAs) are produced in cells of all living organisms^1–3^. Due to absence of free termini, circRNAs are more stable than linear RNAs^4^. Generation of circRNAs usually involves backsplicing in which a downstream 5′ splice site (5′ss) pairs with an upstream 3′ss^5,6^. Biogenesis of circRNAs is a tightly controlled process as backsplicing competes with forward splicing, which is responsible for the production of linear transcripts^7^. Splice site pairing for backsplicing is facilitated by RNA structures as well as by RNA-binding proteins^6^. RNA structures formed by inverted repeat sequences, including Alu elements favor generation of circRNAs^8–10^. Considering Alu elements are present only in primates and are continuing to evolve, their presence within intronic sequences provide species-specific mechanisms for the generation of circRNAs^11^. In some instances, Alu-independent RNA structures enabled by long-range base pairing play pivotal roles in regulation of pre-mRNA splicing, including backsplicing^12^. Functions of circRNAs include sequestration of proteins, sponging of microRNAs, transcription regulation and production of novel proteins^13–16^. CircRNAs are associated with a growing number of pathological conditions and offer novel avenues for diagnosis and therapy^17–19^.

Spinal Muscular Atrophy (SMA) is one of the leading genetic causes of infant mortality^20–23^. In more than 95% of cases, SMA results from the deficiency of the Survival Motor Neuron (SMN) protein due to deletions of or mutations in the *SMN1* gene^20–23^. SMN is involved in many processes, including DNA repair, transcription, translation, mRNA trafficking, stress granule formation and cell signaling^24^. *SMN2*, a near identical copy of *SMN1*, fails to compensate for the loss of *SMN1* due to predominant skipping of exon 7^25,26^. Transcripts lacking exon 7 code for SMNΔ7, a less stable and partially functional protein^27^. Restoration of SMN by promotion of *SMN2* exon 7 inclusion or by gene therapy are two proven approaches for the treatment of SMA^22,28–31^. Deficiency of SMN has also been shown to cause male reproductive organ developmental defects in mice^32^. In contrast, overexpression of SMN has been found to trigger neuroinflammation and the innate immune response in mice^33^. Recently, overexpression of SMN was recorded in patients with laryngeal squamous cell carcinoma (LSCC)^34^. Hence, a tight regulation of SMN appears to be critical for the proper function of the cellular metabolism.

Mechanisms by which the *SMN1* and *SMN2* genes (hereinafter referred to as *SMN1/2*) modulate SMN levels involve both transcription and splicing regulation^35^. About 40% of the sequences of human *SMN1/2* are comprised of Alu elements with the potential to exert unique transcriptional and post-transcriptional regulations^36^. One of the Alu elements of *SMN1*/*2* encompasses the alternatively spliced exon 6B, inclusion of which changes the critical C-terminus of SMN^37^. However, exon 6B of *SMN1*/*2* is generally skipped and exon 6B-included transcripts are subjected to nonsense-mediated decay (NMD)^37^. Consistent with the unusually high number of Alu elements within intronic sequences, human *SMN1*/*2* genes generate a huge repertoire of circRNAs^38,39^. DNA/RNA helicase DHX9 (also known as RHA) and splicing factor Sam68 have been shown to modulate generation of *SMN1*/*2* circRNAs^38,39^. DHX9 is a SMN-interacting protein and is a known regulator of transcription and splicing^40–42^. Sam68 has been previously implicated in regulation of *SMN2* exon 7 splicing^43^. Recent evidence supports that the process of forward splicing and/or the presence of the exon junction complex (EJC) formed during forward splicing favor generation of *SMN1*/*2* circRNAs^44^. Considering pre-mRNAs serve as the source for both circRNAs and mRNAs, generation of *SMN1*/*2* circRNAs may have a role in modulating SMN levels by reducing the levels of *SMN1*/*2* mRNAs. Another way by which *SMN1/2* circRNAs may modulate SMN levels is through sequestration of microRNAs (miRNAs). Additionally, a subset of exonic circRNAs are implied to regulate their parental gene expression at transcriptional, translational or post-translational levels^45^.  However, functions of *SMN1/2* circRNAs remain largely unknown.

**Figure 1:**
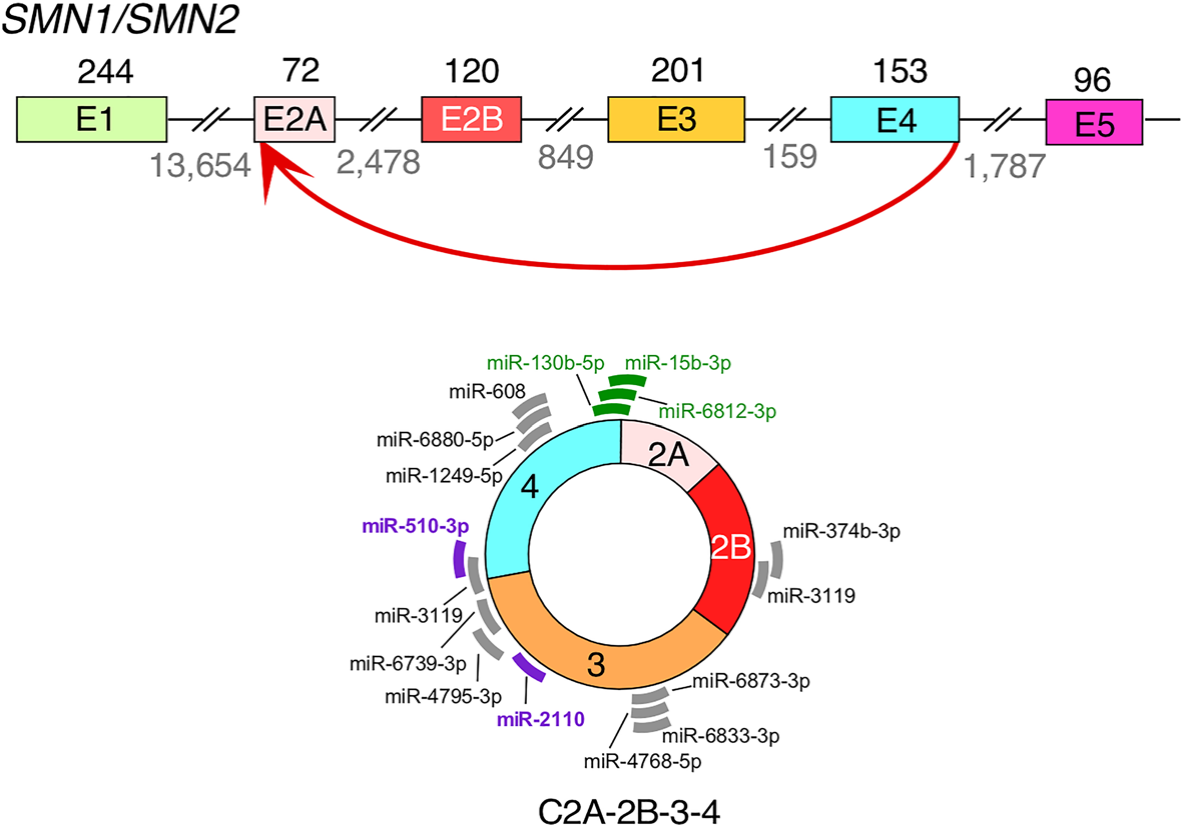
Biogenesis and potential miRNA-sponging activity of *SMN1/2* circRNA C2A-2B-3-4. Top panel: Genomic overview of the early exons of *SMN1*/*2* genes. Exons are depicted as colored shapes and introns as broken lines. Sizes of exons and introns are marked above exons and below introns, respectively. The backsplicing event to form C2A–2B–3–4 is marked with a red arrow. Bottom panel: A diagram of C2A–2B–3–4. Predicted miRNA binding sites are indicated with thick lines^46^. Gray color represents miRNAs of less defined functions. Purple color represents miRNAs with identified functions related to SMN biology and/or neurodegeneration. Green color represents miRNAs whose targets are located across the backsplice junction. Thus, these miRNAs bind differentially to circRNA as compared to their linear mRNA counterparts.

The 546-nucleotide long C2A–2B–3–4 is an abundantly expressed *SMN1*/*2* circRNA encompassing early exons 2A, 2B, 3 and 4 (Fig. 1)^38^. C2A–2B–3–4 is expressed in all human tissues examined, including brain, spinal cord, heart, skeletal muscle, smooth muscle, liver, kidney, lung, uterus and testis^38^. Interestingly, C2A–2B–3–4 is underexpressed in SMA patient fibroblasts^38^. C2A–2B–3–4 is also expressed in mouse cells, underscoring its functional significance across the mammalian kingdom^38^. In addition, identification of C2A-2B-3-4 of *SMN1/2* was reported by another independent study^39^. C2A–2B–3–4 is predicted to interact with at least 16 miRNAs, three of which specifically target the backsplice junction (miR-130b-5p, miR-6812-3p, and miR-15b-3p) and two of which are associated with SMN-related biology and/or neurodegenerative diseases (miR-2110 and miR-510-3p)^46^. We recently reported HEK293-derived inducible stable-cell-lines TC4-2A and TL4-2A that express C2A–2B–3–4 and L2A–2B–3–4, respectively^44^. L2A–2B–3–4 is a linear transcript encompassing exons 2A, 2B, 3 and 4 of *SMN1*/*2*. Here we employ TC4-2A and TL4-2A cells to examine the effect of overexpression of C2A–2B–3–4 and L2A–2B–3–4, receptively, on transcriptome and proteome. Our findings reveal the roles for C2A–2B–3–4 in regulation of transcription, spliceosome function, ribosome biogenesis, lipid metabolism, cytoskeletal formation, and cell proliferation. Expressions of many genes associated with neuronal functions were also affected by C2A–2B–3–4. Our report expands the functions of *SMN1*/*2* genes in regulation of diverse cellular processes.

## Results

### Transcriptome-wide effects of overexpression of C2A–2B–3–4

**Figure 2:**
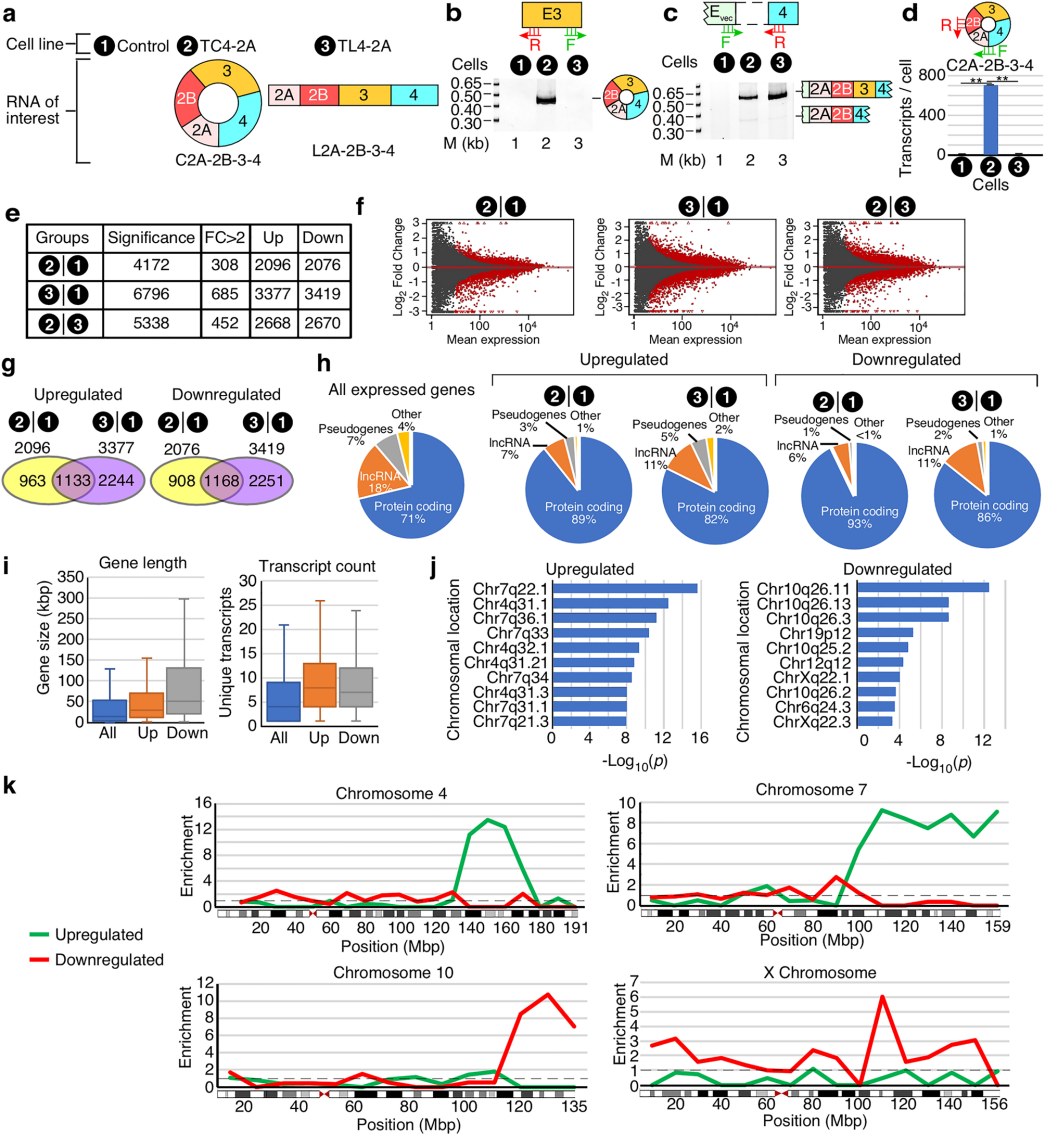
Transcriptome-wide effects of overexpression of *SMN1/2* circRNA C2A–2B–3–4. (**A**) Diagram showing overexpression of C2A–2B–3–4 and L2A–2B–3–4 from TC4-2A and TL4-2A cells, respectively. Control T-REx cells do not overexpress *SMN1*/*2* transcripts. (**B**) A representative gel showing the results of semi-quantitative PCR for detection of circRNA C2A–2B–3–4. Primer annealing sites and cell lines are shown on the top of gel, while lane number is indicated at the bottom. The size marker (M) is indicated on the left side of gel, while the identity of each band on the right side. (**C**) Identification of vector-specific linear transcripts of *SMN1*/*2* generated in different cells. Labeling is the same as in (**B**). (**D**) Quantification of C2A–2B–3–4 expression by qPCR. A diagrammatic representation of C2A-2B-3-4 and primer annealing sites are indicated on the top of bar graph. Error bars represent standard error of the mean. Statistical significance: **, *p* < 0.01. (**E**) Summary of RNA-Seq measuring the impact of overexpression of C2A–2B–3–4 and its linear counterpart L2A–2B–3–4 on the transcriptome. “Significant” indicates genes with Benjamini and Hochberg adjusted *p* value (adj. *p*) < 0.05, out of 19,025 total genes examined for differential expression. “FC > 2” indicates genes with more than twofold up- or downregulation. (**F**) MA plots depicting gene expression changes upon overexpression of C2A–2B–3–4 or L2A–2B–3–4, or a direct comparison between the two. Y-axis: log_2_ fold change. X-axis: mean normalized read counts per gene. Red dots indicate genes with significantly altered expression. (**G**) Venn diagrams examining the overlap between genes altered in the induced TC4-2A and TL4-2A cells compared to control, with upregulated (left panel) and downregulated (right panel) genes indicated. Cell types along with the total number of affected genes are indicated at the top. (**H**) Proportion of protein-coding genes, pseudogenes and lncRNAs among differentially affected genes in TC4-2A and TL4-2A cells compared to T-REx control (**I**) Box plots summarizing the distributions of gene length (left panel) and transcript count (right panel). Boxed area shows the interquartile range (IQR) spanning the middle 50% of values for the given quantification. The median is indicated with a line. Whiskers above and below the box represent upper and lower bound, minus outliers. (**J**) Over-representation analysis (ORA) for specific chromosomal locations of upregulated and downregulated genes. Genomic regions are indicated at the left of each graph. X-axis: − log_10_ of *p* values of enrichment. Left panel examines enrichment in genes upregulated by TC4-2A overexpression, right panel downregulated genes. (**K**) Enrichment by chromosomal position for four chromosomes identified in ORA. Y-axis: enrichment for a given chromosomal region in upregulated (green) or downregulated (red) genes as compared to all genes expressed in T-REx cells. Dashed line indicates enrichment ratio of 1.0, or average enrichment. X-axis: chromosomal position in megabase (Mb). Genes were assigned based on their start position and sorted into bins of 10 Mb. A graphical overview of each chromosome is depicted below each graph. Boxes indicate chromosomal regions, and red triangles indicate centromere position.

To determine the transcriptome-wide effects of overexpression of C2A–2B–3–4, a circRNA harboring *SMN1*/*2* exons 2A, 2B, 3 and 4, we employed the recently reported inducible TC4-2A cell line^44^. For the purposes of comparison, we also employed the inducible TL4-2A cell line that overexpresses L2A–2B–3–4, a linear transcript harboring *SMN1/2* exons 2A, 2B, 3 and 4 (Fig. 2A)^44^. As a control for the background expression profile, we used the parental T-REx cell line without any insertion. In order to capture the broad spectrum of altered cellular activities, overexpression of C2A–2B–3–4 and L2A–2B–3–4 was induced by treatment of the corresponding cell lines with doxycycline (Dox) for 96 hours (h). We validated overexpression by semi-quantitative PCR (Fig. 2B,C). Of note, we used only 22 and 25 cycles of PCR to capture overexpression of C2A–2B–3–4 and L2A–2B–3–4 in TC4-2A and TL4-2A cells, respectively. We also captured linear transcripts (L2A–2B–3–4) as precursors of circular transcripts (C2A–2B–3–4) in TC4-2A cells. In addition, we observed a minor exon 3-skipped transcript L2A-2B-4, which showed a slightly higher expression level in TC4-2A cells than in TL4-2A cells. C2A–2B–3–4 was not detected in either TL4-2A cells or in control T-REx cells at 22 cycles of PCR amplification. To accurately quantify the expression of C2A–2B–3–4, we performed qPCR including a specific primer annealing to the backsplice junction of C2A–2B–3–4 (Fig. 2D). TC4-2A produced an estimated 708 copies of C2A–2B–3–4 per cell, while less than 0.01 copies per cell were produced in TL4-2A and control T-REx cells.

Upon confirming the overexpression of C2A–2B–3–4 and L2A–2B–3–4 in induced TC4-2A and TL4-2A cells, respectively, we characterized the cell growth patterns and morphology of these cells. We used brightfield imaging to capture morphological changes of cells. We did not observe any marked alteration in morphology of cells expressing C2A–2B–3–4 and L2A-2B-3-4, respectively. Cell doubling time of T-REx, TC4-2A and TL4-2A were 22.9, 21.9 and 22.7 h without Dox, and 21.7, 20.8 and 20.9 h with Dox, respectively (Data not shown). Overall, cell doubling time was not overtly changed by C2A–2B–3–4 or L2A–2B–3–4 overexpression, except for a slight stimulatory effect of Dox treatment on cell growth. Next, to characterize the effect of C2A–2B–3–4 and L2A–2B–3–4 on the transcriptome, we performed RNA-Seq. Compared to the control T-REx cells, TC4-2A and TL4-2A cells showed altered expression of 4172 and 6796 transcripts, respectively, with 308 and 685 genes undergoing major changes > twofold, respectively (Fig. 2E). As a frame of reference, we analyzed 26,828 genes with measurable expression in T-REx cells, meaning ~ 16% and ~ 25% of transcripts were affected in TC4-2A and TL4-2A cells, respectively. Altered expression was evenly split between upregulated and downregulated transcripts (Fig. 2E,F). ~ 55% of genes affected in TC4-2A cells were similarly affected in TL4-2A cells (Fig. 2G). Among upregulated genes, this amounted to a 4.3-fold enrichment compared to random chance as calculated by a hypergeometric test (*p* = 2.4 × 10^−516^). For downregulated genes, there was a 4.4-fold enrichment (*p* = 7.6 × 10^−554^). The remaining 45% genes with altered expressions were unique to TC4-2A cells (Fig. 2G). Direct comparison between transcripts of TC4-2A and TL4-2A cells showed altered expressions of 5338 genes (Fig. 2E,F). We observed higher proportion of protein-coding genes affected in both TC4-2A and TL4-2A cells, with lncRNA and pseudogenes under-represented in both upregulated and downregulated genes compared to the parent set of 26,828 genes expressed in T-REx cells (Fig. 2H).

For the remainder of our analyses, we focused on the genes specifically affected in TC4-2A cells overexpressing C2A–2B–3–4. Upregulated genes were longer on average, with a median size of 30.4 kilobases (kb) compared to 14.8 kb for all genes expressed in T-REx cells (Fig. 2I, left panel). Downregulated genes were even longer, with a median size of 50.5 kb. Affected genes had higher transcript complexity with a median unique transcript count of 8 and 7 for upregulated and downregulated genes, respectively, compared to 4 for all genes (Fig. 2I, right panel). There was a marked enrichment in upregulated genes for several regions in chromosomes 4 and 7, and for downregulated genes in several regions of chromosomes 10 and X (Fig. 2J). We mapped the genes identified by RNA-Seq by chromosomal location and calculated enrichment throughout chromosomes 4 and 7 (Fig. 2K). We found 61 upregulated genes located between 135 megabase (Mb) and 160 Mb on chromosome 4, representing ~ 46% of all expressed genes in the same region, a 12.9-fold enrichment over a random distribution. There were no downregulated genes in the same region of chromosome 4. We identified 187 upregulated genes located between 90 Mb and the end of the chromosome 7 (~ 159 Mb), representing ~ 28% of all expressed genes in the same region, a 7.8-fold enrichment. Downregulated genes in this region of chromosome 7 were under-represented by 3.2-fold. There were 62 downregulated genes from 110 Mb to the end of chromosome 10 (~ 133 Mb), representing ~ 30% of all expressed genes located in the same region, a ninefold enrichment (Fig. 2J,K). There were no upregulated genes in the same region of chromosome 10. We observed moderate enrichment of downregulated genes across the entire X chromosome (Fig. 2K). Specifically, there were 65 downregulated genes, ~ 8% of all X-linked genes, representing 2.3-fold enrichment. Upregulated genes were under-represented by 1.9-fold. The region from 100 to 115 Mb of the X chromosome was the most highly enriched, which harbored 26 downregulated genes. This accounted for 20% of all expressed genes in this region, a sixfold enrichment. Upregulated genes in this same region were under-represented by ~ 1.5-fold.

### Gene ontology terms and pathways enriched in genes affected by overexpression of C2A–2B–3–4

**Figure 3:**
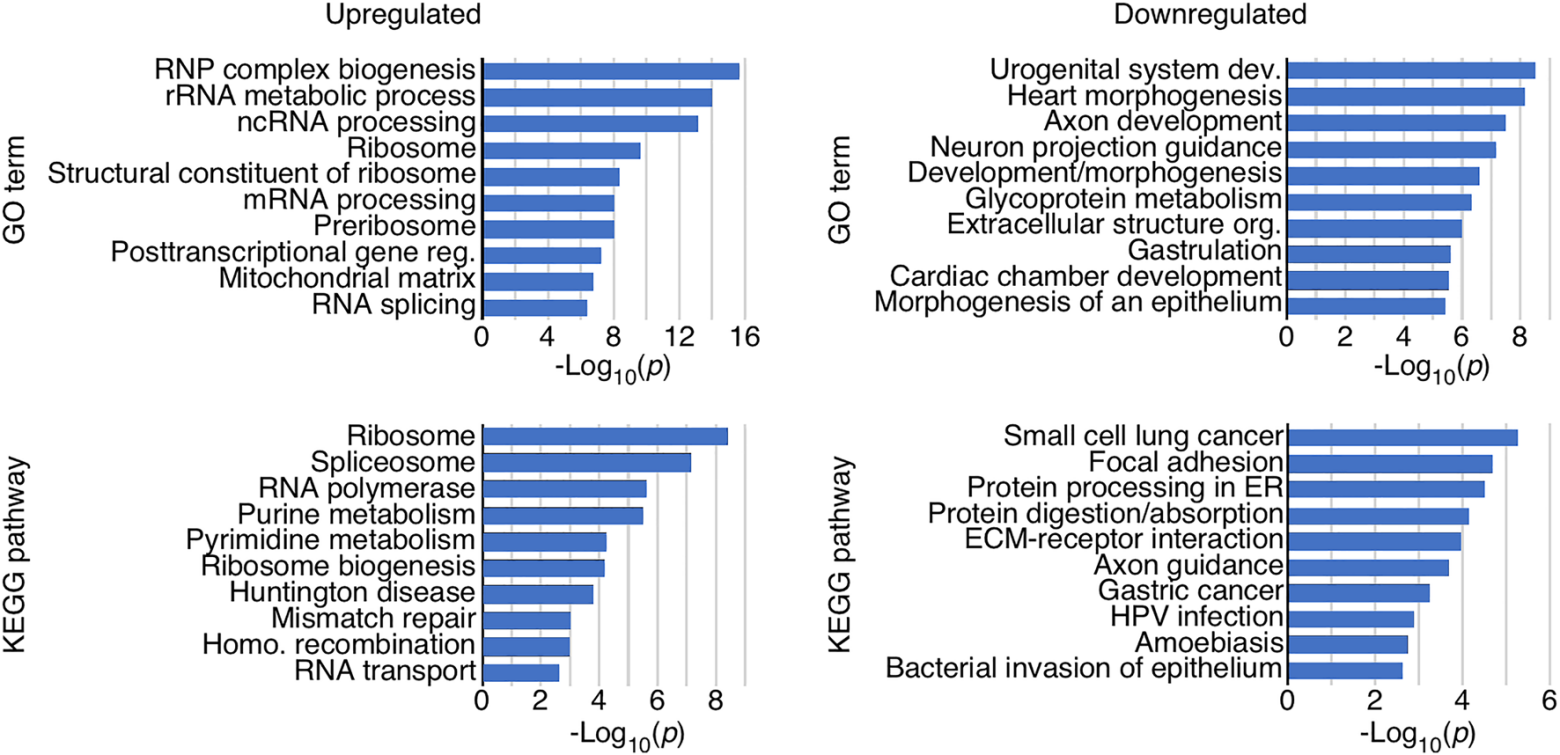
GO terms and pathways enriched among genes affected by C2A-2B-3-4 overexpression. ORA of specific GO terms (top panels) and pathways (including KEGG, Panther, Reactome and Wikipathways) (bottom panels) among genes upregulated (left panels) and downregulated (right panels) by C2A-2B-3-4 overexpression. Categories of genes are indicated at the left of each graph. X-axis: − log_10_ of *p* values of enrichment.

Gene ontology (GO) terms group genes by functional category within three groupings: biological process, cellular component, and molecular function. Genes upregulated in TC4-2A cells overexpressing C2A–2B–3–4 were enriched for several GO terms related to RNA regulation and ribonucleoprotein (RNP) biogenesis, suggesting a major shift in RNA regulation and metabolism (Fig. 3). In contrast, genes downregulated in TC4-2A cells were enriched for GO terms related to the extracellular matrix and cellular morphology (Fig. 3). We also analyzed genes for enrichment in functional pathways as defined by the Kyoto Encyclopedia of Genes and Genomes (KEGG), Panther, Reactome and Wikipathways. Upregulated genes were enriched for several pathways involved in RNA regulation, including RNA polymerase, spliceosome, ribosome, and RNA transport. We observed an enrichment in the pathways for homologous recombination and DNA mismatch repair, suggesting an effect on DNA maintenance (Fig. 3). Downregulated genes were enriched for pathways involving protein processing, endoplasmic reticulum (ER), and cell morphology and motility.

### qPCR validation of a broad spectrum of genes upregulated by overexpression of C2A–2B–3–4

**Figure 4:**
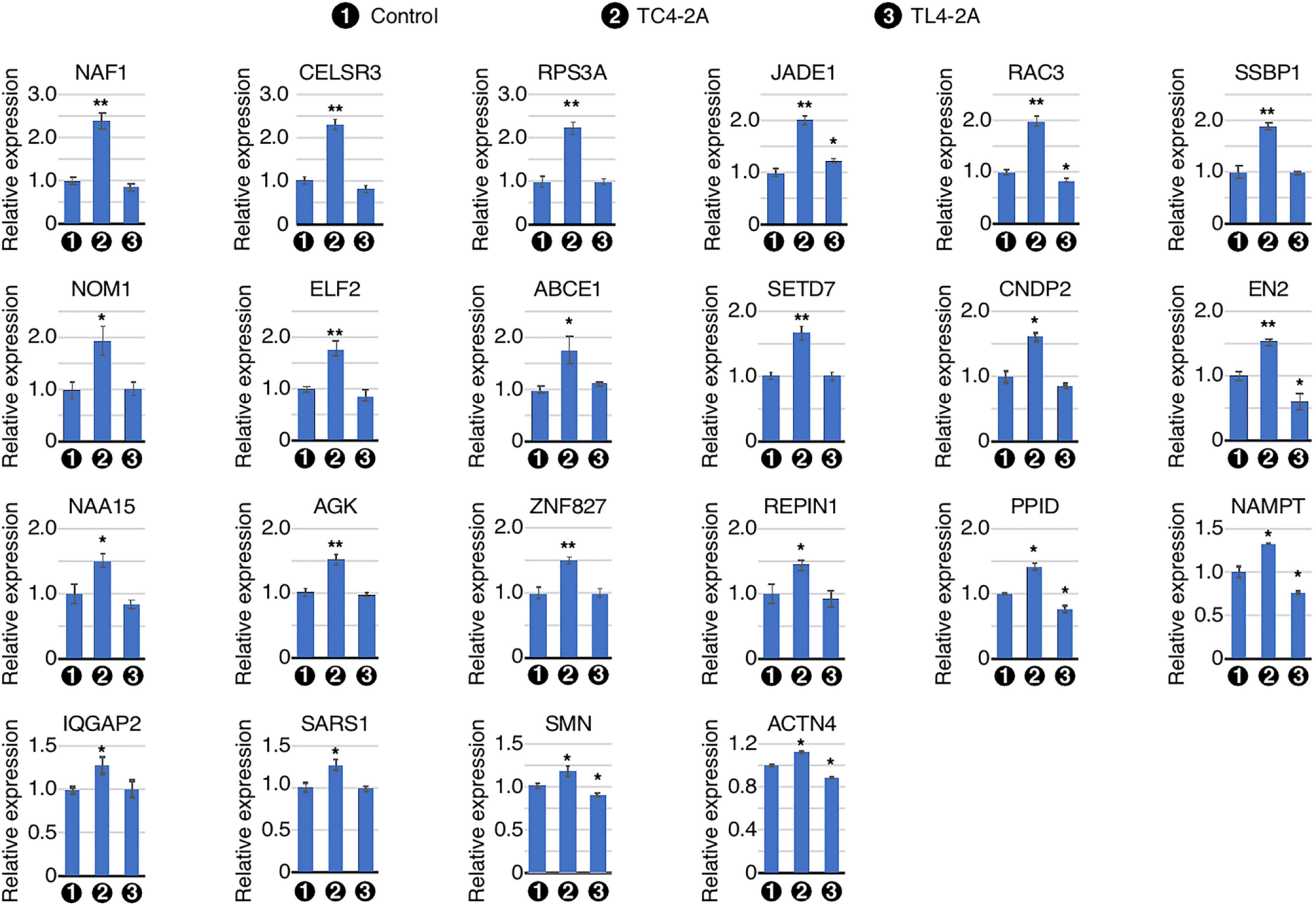
qPCR validation of transcripts predicted by RNA-seq to be upregulated upon C2A-2B-3-4 overexpression. Bar graphs represent transcript levels of the indicated genes in RNA isolated from Dox-treated cells, including control T-REx control, TC4-2A and TL4-2A cells, as measured by qPCR. The gene symbol is indicated above each graph. Cell types are indicated under the X-axis. The Y-axis represents the relative expression as compared with control T-REx cells. Error bars represent standard error of the mean. Statistical significance: n = 3 *, p < 0.05; **, p < 0.01.

We independently confirmed the results of RNA-Seq by qPCR. First, we validated the upregulation of eight genes located in the enriched region of chromosome 4, namely *NAF1, RPS3A*, *ELF2*, *ABCE1*, *SETD7*, *NAA15*, *ZNF827*, and *PPID* (Fig. 4). *NAF1* codes for a box H/ACA RNP biogenesis factor. *NAF1* was upregulated ~ 2.4-fold in TC4-2A cells. We confirmed a ~ 2.2-fold increase in expression of the *RPS3A* small ribosomal subunit gene in TC4-2A cells. *ELF2* codes for a member of the Ets family of transcription factors; its expression was increased about ~ 1.8-fold in TC4-2A cells. ABCE1 mediates the dissociation of eukaryotic post-termination complexes into free ribosomal subunits. We captured a ~ 1.8-fold increase in the expression of *ABCE1* in TC4-2A cells. We confirmed a ~ 1.7-fold upregulation of the H3K4 histone lysine methyltransferase *SETD7* in TC4-2A cells. *NAA15* codes for an auxiliary subunit of the N-terminal acetyltransferase A (NatA) complex; its expression was increased by ~ 1.5-fold in TC4-2A cells. ZNF827 is a zinc finger protein involved in telomere maintenance, homologous recombination, and the epithelial to mesenchymal transition. We observed a ~ 1.5-fold upregulation of *ZNF827* in TC4-2A cells. *PPID* codes for a molecular chaperone that regulates protein folding; its expression was found to be increased by ~ 1.4-fold in TC4-2A cells. Interestingly, we noted downregulation of *PPID* in TL4-2A cells, suggesting that C2A–2B–3–4 and L2A–2B–3–4 have opposite effects on its expression (Fig. 4).

We validated the C2A–2B–3–4-induced upregulation of six genes located in the right arm of chromosome 7. These were *SSBP1*, *NOM1*, *EN2*, *AGK*, *REPIN1*, and *NAMPT*. Our results showed a ~ 1.9-fold increase in expression of the mitochondrial DNA synthesis factor *SSBP1* in TC4-2A cells (Fig. 4). *NOM1* encodes a nucleolar protein NOM1 that participates in rRNA biogenesis. We observed a ~ twofold upregulation of *NOM1* in TC4-2A cells. We captured a ~ 1.5-fold increase in expression of transcripts encoding the homeobox transcription factor EN2 in TC4-2A cells. *AGK* encodes a mitochondrial membrane protein associated with lipid and glycerolipid metabolism. *AGK* was found to be upregulated by ~ 1.5-fold in TC4-2A cells. We observed a ~ 1.4-fold upregulation of *REPIN1*, which codes for a zinc finger DNA-binding protein that regulates DNA replication, in TC4-2A cells. *NAMPT* encodes a regulator of intracellular nicotinamide adenine dinucleotide (NAD) metabolism. Expression of *NAMPT* was upregulated by ~ 1.3-fold in TC4-2A cells, while its expression was reduced by ~ 24% in TL4-2A cells.

We also measured expression of several upregulated genes outside of the enriched genomic regions. CELSR3 is required to regulate cell polarity and neuronal axon guidance and wiring in multiple brain regions and neuronal cell types. We observed a ~ 2.3-fold increase in expression of *CELSR3* TC4-2A cells (Fig. 4). *JADE1* encodes a scaffolding protein that regulates histone acetyltransferase subunit HBO1. We captured a ~ twofold upregulation of *JADE1* in TC4-2A cells, while its expression was slightly (~ 1.2-fold) increased in TL4-2A cells. *RAC3* codes for a GTPase that belongs to the RAS superfamily of small GTP‑binding proteins. We recorded a twofold increase in expression of *RAC3* in TC4-2A cells, while its expression was slightly reduced in TL4-2A cells. *CNDP2* controls turnover of carnosine and other dipeptides. Expression of *CNDP2* was increased by ~ 1.6-fold in TC4-2A cells. We observed a ~ 1.3-fold upregulation of *IQGAP2*, which codes for a scaffolding protein involved in cytoskeleton regulation, in TC4-2A cells. Seryl tRNA synthestase gene *SARS1* was found to be upregulated by ~ 1.3-fold in TC4-2A cells. *ACTN4* codes for actin crosslinking protein alpha-actinin-4. *ACTN4* showed a moderate (~ 1.1-fold) upregulation in TC4-2A cells, while it was slightly downregulated in TL4-2A cells. We also measured transcript levels of *SMN1*/*2*. Interestingly, we captured a ~ 1.2-fold increase in expression of *SMN1*/*2* in TC4-2A cells, while *SMN1*/*2* expression was reduced by ~ 10% in TL4-2A cells (Fig. 4). Our results suggested that the circular and linear transcripts of *SMN1*/*2* encompassing exons 2A, 2B, 3 and 4 have positive and negative effects, respectively, on expression of linear transcripts of *SMN1*/*2*. Of note, qPCR amplification of *SMN1*/*2* transcripts does not distinguish among different linear isoforms of transcripts generated by *SMN1*/*2.*

### qPCR validation of a panel of genes downregulated by C2A–2B–3–4 overexpression

**Figure 5:**
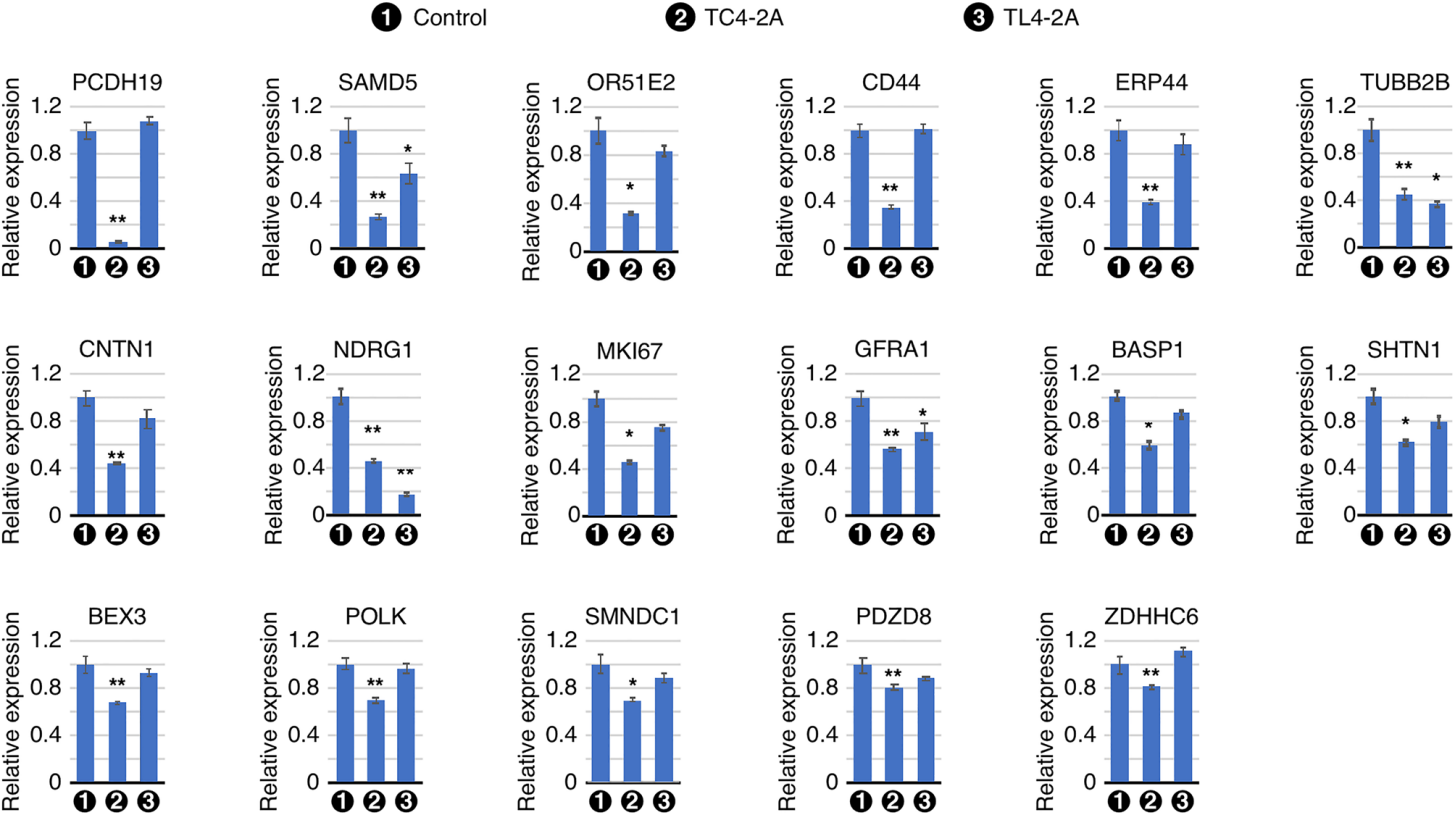
qPCR validation of transcripts predicted by RNA-seq to be downregulated upon C2A-2B-3-4 overexpresion. Bar graphs show transcript levels of the indicated genes in RNA isolated from Dox-treated T-REx control, TC4-2A and TL4-2A cells, as measured by qPCR. Labeling is the same as in Fig. 4.

By employing qPCR, we confirmed downregulation of genes captured by RNA-Seq of transcripts isolated from TC4-2A cells. We began with validation of six genes located in the enriched region of chromosome 10. These were *MKI67*, *GFRA1*, *SHTN1*, *SMNDC1*, *PDZD8*, and *ZDHHC6* (Fig. 5). *MKI67* codes for a marker of cellular proliferation that is associated with multiple cancers. We observed a > 54% reduction in expression of *MKI67* in TC4-2A cells (Fig. 5). The expression of *MKI67* appeared to be also reduced in TL4-2A cells, however, the change was not statistically significant. We captured reduced expression of GDNF co-receptor gene *GFRA1* in both TC4-2A and TL4-2A cells, although the response was slightly stronger in TC4-2A cells (Fig. 5). *SHTN1* codes for SHOOTIN1, an actin-binding protein involved in axon growth. We recorded a ~ 38% reduction in expression of *SHTN1* in TC4-2A cells. We observed a ~ 30% reduction in expression of *SMN* paralog *SMNDC1*, which functions in splicing, in TC4-2A cells. *PDZD8* and *ZDHHC6* were both downregulated by ~ 19% in TC4-2A cells. We validated expression of two X-chromosome linked genes, *PCDH19* and *BEX3*, that were captured by RNA-Seq to be downregulated by C2A–2B–3–4. *PCDH19* codes for a calcium‑dependent cadherin with preferential expression in brain. We confirmed a > 16-fold reduction in expression of *PCDH19* in TC4-2A cells, while no effect was observed in TL4-2A cells. We observed ~ 32% lower expression of transcriptional regulator *BEX3* in TC4-2A cells.

We validated expression of additional important genes that were captured by RNA-Seq to be downregulated in TC4-2A cells. These were *SAMD5*, *OR51E2*, *CD44*, *ERP44*, *TUBB2B*, *CNTN1*, *NDRG1*, *BASP1*, and *POLK*. *SAMD5* encodes a SAM-domain containing protein of less defined function; we observed a ~ 3.7-fold reduction in its expression in TC4-2A cells, while it was decreased by ~ 37% in TL4-2A cells (Fig. 5). *OR51E2* encodes an olfactory receptor that also plays a role in the gut. We captured a ~ threefold downregulation of *OR51E2* in TC4-2A cells. Protein encoded by *CD44* mediates several functions, including cell–cell interaction, cell adhesion and migration. We recorded a ~ 2.9-fold lower expression of *CD44* in TC4-2A cells. There was a ~ 60% reduction in expression of transcripts coding for the ER redox sensor protein ERP44 in TC4-2A cells. *TUBB2B* encodes a beta tubulin protein that is mutated in polymicrogyria, a cortical development disorder. *TUBB2B* was reduced  by > twofold in both TC4-2A and TL4-2A cells (Fig. 5). CNTN1, a neuronal membrane protein, belongs to the immunoglobulin superfamily. We observed a ~ 2.2-fold reduction of expression of *CNTN1* in TC4-2A cells (Fig. 5). *NDRG1* encodes a universally expressed protein that plays a role in myelin sheath formation. We observed a ~ 2.2-fold decrease in expression of *NDRG1* in TC4-2A cells. Interestingly, expression of *NDRG1* was reduced by > fivefold in TL4-2A cells (Fig. 5). BASP1 participates in axonal growth, regeneration, and plasticity. We captured a ~ 40% reduction in expression of *BASP1* is in TC4-2A cells. The expression of transcript coding for DNA repair-associated polymerase POLK was reduced by > 30% in TC4-2A cells.

### Effect of overexpression of C2A–2B–3–4 on the cellular proteome

**Figure 6:**
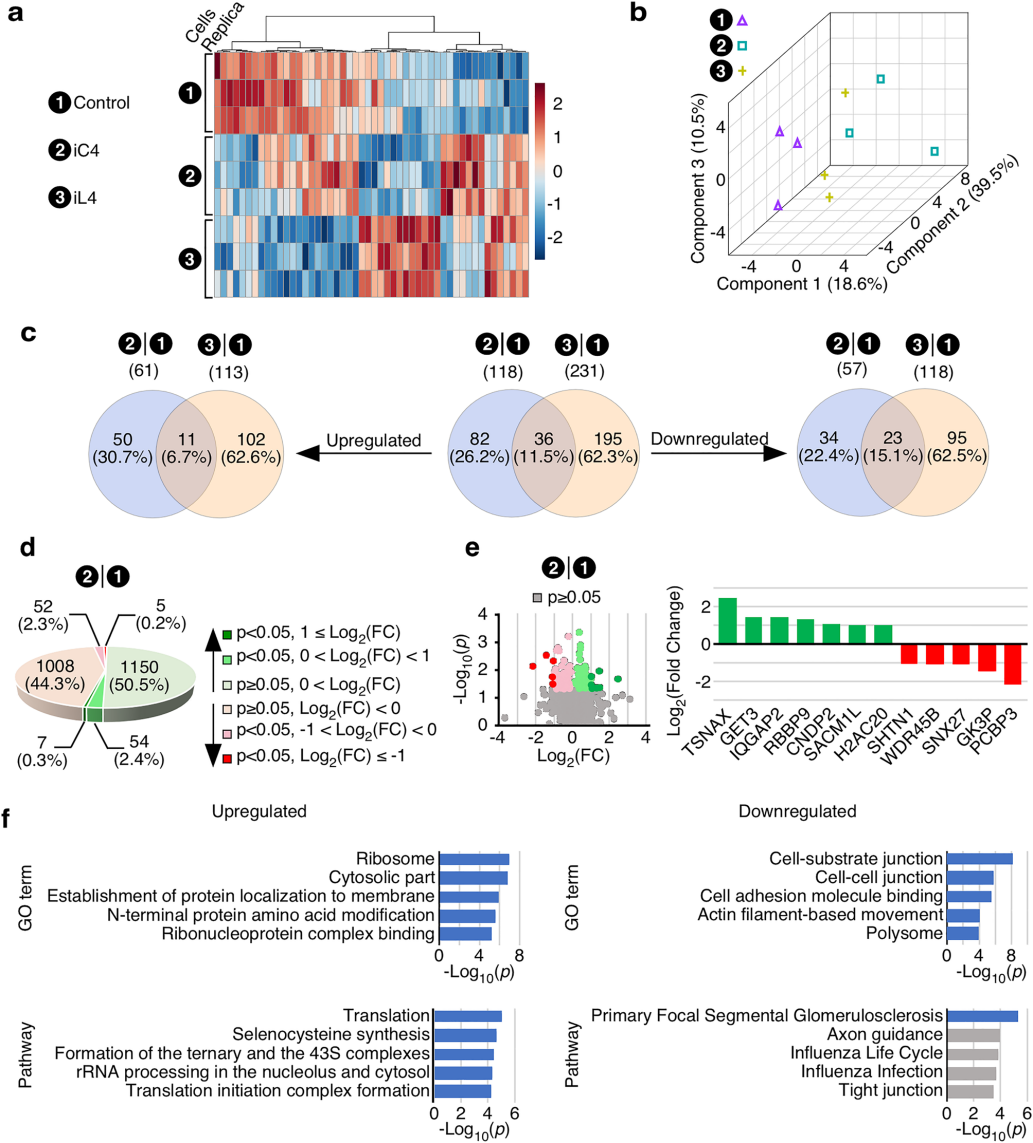
Effects of overexpression of C2A–2B–3–4 and L2A–2B–3–4 on the proteome of human cells. (**A**) A heatmap generated by hierarchical cluster analysis of each individual sample used for proteomic analysis across three cell lines. The cell lines are indicated on the left side of the heat map. The top 50 proteins are shown. The Euclidean distance was used as similarity measuring parameter, while Ward’s linkage was the clustering algorithm. Each colored square indicates relative abundance of an identified protein. Cell line and replicate was marked on the left of heatmap. On the right side showing a color key (high, red; low, blue) indicates the scale of relative abundance determined by Euclidean distance. (**B**) A three-dimensional (3D) scores plot of Partial Least-Squares Discriminant Analysis (PLS-DA) of each individual sample. The top three most discriminant components were selected to plot data in three directions. Each individual replicate is indicated with a colored shape. (**C**) Venn diagrams showing the overlap between proteins significantly altered (*p *< 0.05) in the TC4-2A and TL4-2A cells compared to control, with total affected (center panel), upregulated (left panel) and downregulated (right panel) proteins indicated. Cell lines and total number of affected proteins are indicated at the top. (**D**) An overview of proteins identified in TC4-2A (Supplementary Table S4). Proteins are grouped based on regulation direction and statistical significance. The number of proteins identified in each group are indicated, while the percentage of the total identified proteins is shown in parentheses. Color coding is indicated on the right of pie graph. (**E**) Identities of the most significantly altered proteins in TC4-2A. Left panel: Volcano plot of all identified proteins in TC4-2A. The X-axis indicates log_2_ transformed value of fold change (FC), whereas the Y-axis indicates the -log_10_ transformed *p* value. Each individual protein was plotted as a colored dot: grey indicates proteins showing *p* ≥ 0.05, other color-coding is the same as in (**D**). Right panel: Log_2_(FC) of expression levels of the proteins most strongly affected by C2A-2B-3-4 overexpression. The protein names are labeled under X-axis. Y-axis indicates log_2_ value of FC. Upregulated and downregulated proteins are shown in green and red, respectively. (**F**) ORA of GO terms (top panel) and pathways (bottom panel) significantly upregulated (left panel) and downregulated proteins (right panel) in TC4-2A, respectively.

To profile the changes in the cellular proteome caused by overexpression of C2A–2B–3–4 and L2A–2B–3–4, we performed label-free relative quantification of protein expression. We performed these experiments using exactly the same three groups of cells (control T‑REx,

TC4-2A and TL4-2A cells) that were utilized for RNA-Seq. We identified 3818 expressed proteins with an FDR of 0.05. We analyzed the overall composition of the three proteomes using a heatmap of the 50 proteins with the highest divergence between sample groups. As expected, the three groups of cells showed distinctive expression patterns (Fig. 6A). This was further confirmed by using principal component analysis (Fig. 6B). The three groups cluster separately, although there were similarities between TC4-2A and TL4-2A cells. After implementing data integrity check, filtering, and normalization, we performed differential expression analysis including 2276 and 2279 proteins in TC4-2A and TL4-2A cells by comparing to control T‑REx cells, respectively. We identified 118 and 231 significantly altered proteins in TC4-2A and TL4-2A cells, respectively (Fig. 6C–E). More than 69% and 84% of altered proteins were unique to TC4-2A and TL4-2A, respectively, however there was significant overlap. Specifically, of 118 proteins that were altered in TC4-2A cells, 82 were unique and 36 were shared with TL4-2A cells, while 195 proteins were affected in TL4-2A cells alone (Fig. 6C). The proteins upregulated by each group were more distinct, with 50 unique to TC4-2A cells, 11 shared between TC4-2A and TL4-2A cells, and 102 unique to TL4-2A cells. The inverse was true of downregulated proteins with 34 unique to TC4-2A cells, 23 were shared between TC4-2A and TL4-2A cells, and 95 unique to TL4-2A cells. Of note, there are two proteins (SUB1 and YBX3) that were regulated in opposite directions in TC4-2A and TL4-2A, respectively. Hence, in each cell line, subset size varies by 2 when comparing the sum of the subsets (uniquely affected or overlapped) of up- (left panel) and downregulated (right panel) proteins to that of the summarizing panel shown in the center.

To examine the changes caused by overexpression of C2A-2B-3-4 further, we focused on proteins altered in TC4-2A as compared to control T-REx cells. Among 2276 proteins identified in TC4-2A, slightly more proteins were upregulated. There were 61 proteins were significantly upregulated and 57 were significantly downregulated, with the expression ~ 10% of proteins being altered > twofold (Fig. 6D,E, Supplementary Table S4). The most significantly upregulated proteins were TSNAX, GET3, IQGAP2, RBBP9, CNDP2, SACM1L and H2AC20 (Fig. 6E). TSNAX, participating in RNA silencing, was the most upregulated protein in TC4-2A cells, increasing by ~ 5.5-fold. GET3, an ATPase that targets future tail-anchored membrane proteins to the ER, was the second most upregulated protein with a ~ 2.8-fold increase. IQGAP2, a scaffolding protein involved in cytoskeleton regulation, was also captured in our RNA-Seq analysis. IQGAP2 was upregulated by ~ 2.7-fold in TC4-2A cells. RBBP9, a serine hydrolase mainly localized in nucleoplasm, was upregulated by ~ 2.5-fold in TC4-2A cells. CNDP2, a carnosine dipeptidase, was also identified in our RNA-Seq analysis and qPCR validation. The expression of CNDP2 was elevated by ~ 2.1-fold in TC4-2A cells. SACM1L is a phosphatidylinositol phosphatase that plays a role in autophagosome formation. SACM1L was upregulated by ~ twofold in TC4-2A cells. The expression of histone H2A family member H2AC20 was increased by ~ twofold in TC4-2A cells.

The five most significantly downregulated proteins in TC4-2A were PCBP3, GK3P, SNX27, WDR45B, and SHTN1 (Fig. 6E). Multi-functional nucleic acid binding protein PCBP3 was the most downregulated protein, with a ~ 4.5-fold change in TC4-2A cells compared to control. GK3P (also known as GK3) is a glycerol kinase involved in general energy metabolism. GK3P, coded by *GK3*, was downregulated by ~ 2.7-fold in TC4-2A cells. The *GK3* gene is unspliced and located in the intron of another gene, *KLHL2*. Therefore, it may be a pseudogene. Consistently, we did not find many RNA-Seq reads mapping to the *GK3* gene. However, the *GK* gene is predicted to be downregulated ~ twofold by our RNA-Seq analysis and qPCR validation (Supplementary Fig. S4), while *GK3* was upregulated by > 1.7-fold in qPCR validation. It is possible that similarity between peptides produced by GK and GK3P caused the proteomics analysis software to mistakenly assign GK peptides to GK3P. Vesicle sorting protein SNX27 was downregulated by ~ 2.2-fold. We observed a ~ 2.1-fold downregulation of autophagy-related protein WDR45B in induced TC4-2A cells. Actin-binding protein SHTN1 was downregulated by ~ 2.1-fold in TC4-2A cells. Incidentally, upregulation of *SHTN1* in TC4-2A cells was also captured in RNA-Seq analysis and validated by qPCR.

We analyzed the significantly altered proteins in TC4-2A cells for enrichment of GO terms and pathways. We focused on top 5 most significant events with FDR < 0.05 (Fig. 6F and Supplementary Fig. S1). Consistent with the findings of RNA-Seq, significantly upregulated proteins in TC4-2A cells were associated with translation, ribosome activity, localization of RNA and proteins, and protein modification. Significantly downregulated proteins in TC4-2A cells were mainly related to cytoskeletal activity, cell adhesion, and regulation of extracellular proteins. We also analyzed enrichment of proteins altered in TL4-2A cells compared to control cells and TC4-2A cells compared directly to TL4-2A cells (Supplementary Figs. S2, S3). Like TC4-2A cells, TL4-2A cells had upregulation of ribosome components and translation-related proteins (Supplementary Fig. S2). However, the enriched GO terms and pathways of downregulated proteins in TL4-2A were fully distinct.

### Validation of proteins affected by overexpression of C2A–2B–3–4

**Figure 7:**
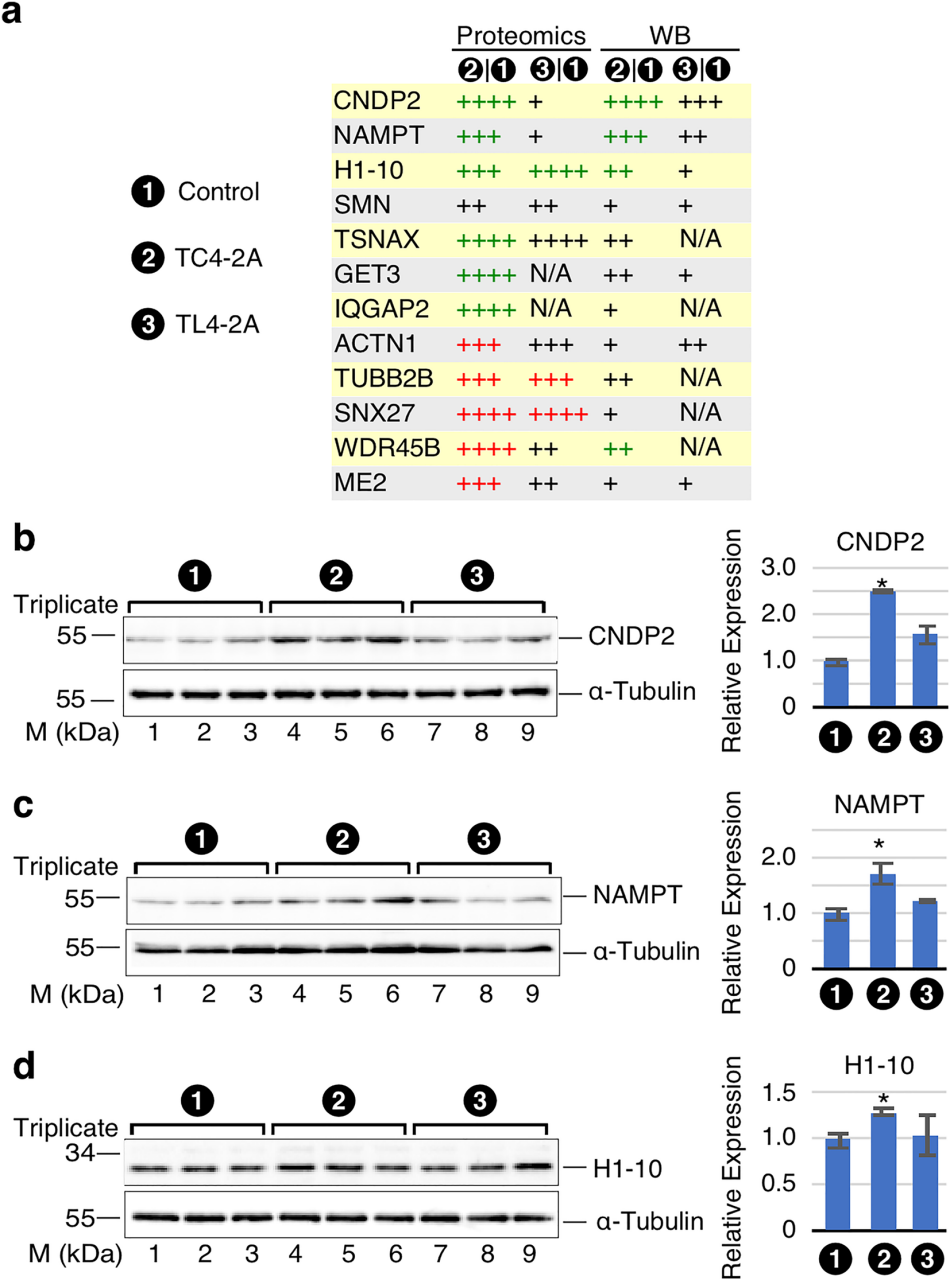
Western blot validation of differentially expressed candidates identified by proteomics analysis of C2A–2B–3–4 overexpression. (**A**) Expression profiles of top candidates identified in proteomics analysis. Expression levels are summarized as follows: FC < 1.2, +; ≥ 1.2, ++; ≥ 1.5, +++; ≥ 2, ++++, while green and red color indicate up- and downregulation, respectively, with *p* or adj. *p* < 0.05. (**B**–**D**) Western blot results of validated candidates. Left panel: Representative blots. α-tubulin is used as loading control. Cell line is indicated at the top of the panel. Antibody used is indicated at the right and nearby molecular weight markers are indicated at the left. Right panel: quantification of western blot results. For each band, background signal was subtracted and then signal was normalized by α-tubulin. Error bars represent standard error of the mean (SEM). n = 3, *p < 0.05 compared to T-REx control.

To validate the findings of proteomics, we performed western blot for 14 representative candidates affected in TC4-2A cells (Fig. 7A, Supplementary Fig. S4). Our results confirmed the expected upregulation of three proteins, including CNDP2, NAMPT and H1-10 (Supplementary Table S5). For instance, we observed a ~ 2.5-fold increase in expression of CNDP2 in TC4-2A cells (Fig. 7B). NAMPT (also known as PBEF) was upregulated by ~ 1.7-fold in TC4 (Fig. 7C). We recorded a ~ 1.3‑fold increase of H1‑10 (also known as H1X) (Fig. 7D). For many proteins, including SMN, we could validate the trend towards upregulation in TC4-2A cells, but the observed changes were not statistically significant (Supplementary Fig. S4). This is likely due to the fact that changes captured by proteomics are driven by peptides derived from different protein isoforms not well-recognized by the available antibodies. Also, western blot may not be sensitive enough to accurately capture small changes in protein levels. By cross-examining the proteomics and RNA-Seq analysis, multiple candidates, and their coding genes with significantly altered expression were identified in both analyses. These genes included *CNDP2*, *NAMPT*, *IQGAP2* and *TUBB2B* (Supplementary Table S5).

## Discussion

We examined the effects of overexpression of C2A–2B–3–4, a ubiquitously expressed circRNA encompassing exons 2A, 2B, 3 and 4 of *SMN1*/*2* on the transcriptome and proteome of human cells. In parallel, we also examined the effects of  overexpression of L2A–2B–3–4, a linear transcript encompassing the entire sequence of C2A–2B–3–4 but in a forward manner. The objective of our study was to capture the circRNA-specific effects that cannot be exerted by identical sequences in various mRNA isoforms of *SMN1*/*2*. Overexpression of C2A–2B–3–4 and L2A–2B–3–4 was induced by Dox treatment of HEK293-derived TC4-2A and TL4-2A cells stably expressing C2A–2B–3–4 and L2A–2B–3–4, respectively. We captured a significant impact of C2A–2B–3–4 on transcriptome as expressions of ~ 15% of all detectable genes. Nearly half of the affected genes by C2A–2B–3–4 were unique as they were not impacted by L2A–2B–3–4. The specific effect of C2A–2B–3–4 could be attributed to multiple factors, including the presence of unique backsplice junction sequence, distinct secondary and tertiary structures^47^ and m^6^A modifications of circRNAs^48,49^. About 55% of genes affected by C2A–2B–3–4 overlapped with those affected by L2A–2B–3–4. The overlapping effects of C2A–2B–3–4 and L2A–2B–3–4 could be due to common sequence motifs that are not structurally constrained. The specific effects on transcriptome captured with L2A–2B–3–4 but not with C2A–2B–3–4 could be attributed to the structurally unconstrained sequence motifs present exclusively within L2A–2B–3–4.

C2A–2B–3–4 specifically affected large protein-coding genes with higher complexity defined by transcripts undergoing alternative splicing and/or utilizing multiple 5′ untranslated regions (5′UTRs) or 3′UTRs (Fig. 2). There was a striking enrichment of upregulated genes in the large arms of chromosomes 4 and 7, suggesting that C2A–2B–3–4 promotes formation of extended open chromatin structures in the upregulated regions of chromosomes 4 and 7. Opening of the chromatin structure by C2A–2B–3–4 could be brought about by DNA hypomethylation similarly as reported in case of the exon-containing circRNAs expressed from *FLI1* gene^50^. We observed a significant enrichment of downregulated genes in the extended regions of chromosomes 10 and X, suggesting formation of compact chromatin states in the downregulated regions of these chromosomes. Promotion of repressed state of chromatin may involve factors that favor formation and/or extension of heterochromatin. Exonic circRNAs can also regulate transcription by inhibiting the activity of transcription factors^45^. Expression of genes located on other chromosomes were also impacted by C2A–2B–3–4. Genes that were upregulated by C2A–2B–3–4 were highly enriched for RNA biogenesis and RNA processing factors, especially those associated with the ribosome and spliceosome function (Fig. 3). Downregulated genes were enriched for ER proteins and pathways involved in cell and organ development, morphology, and maintenance of extracellular proteins (Fig. 3). SMN plays an important role in development and maintenance of axonal growth^51–54^. It is possible that the axonal defects observed in SMA could be attributed at least in part to the dysregulation of C2A–2B–3–4.

Several of the upregulated genes that we validated by qPCR code for important proteins, including H/ACA RNP biogenesis factor (*NAF1*), ribosomal component (*RPS3A*), ribosome associated factors (*ABCE1* and *NOM1*), tRNA synthetase (*SARS1*), transcription factors (*ELF2*, *EN2* and *ACTN4*), chromatin modifiers (*SETD7*, *NAA15* and *JADE1*), telomerase associated factor (*ZNF827*), protein folding regulator (*PPID*), mitochondrial factors (*SSBP1* and *AGK*), DNA replication factor (*REPIN1*), NAD metabolism regulator (*NAMPT*), neuronal exon guidance regulator (*CELSR3*), and signaling factors (*RAC3*, *CNDP2* and *IQGAP2*). Several alternatively spliced transcripts are generated by *SMN*1/2^55^. Interestingly, *SMN1/2* transcripts were upregulated and downregulated by C2A–2B–3–4 and L2A–2B–3–4, respectively (Fig. 4). However, our analysis did not distinguish among different alternatively spliced transcripts of *SMN1/2*. Growing evidence suggest that the expression of genes could be regulated by their own circRNAs through direct recruitment of RNA polymerase (pol II) and/or recruitment of transcription and chromatin remodeling factors as well as by regulation of R-loop formation^45^. Such regulation of transcription could have a cascading effect on the nearby genes. Consistently, we captured the effect of C2A–2B–3–4 on transcription of *IQGAP2* and *POLK* situated in the vicinity of *SMN1/2* locus. However, these results should be interpreted with caution as C2A–2B–3–4 is expressed from a different locus in the stable cell line we used. Our finding of upregulation of *SMN1*/*2* transcripts by C2A–2B–3–4 expressed from a different locus supports a hypothesis that C2A–2B–3–4 serves as a sensor to regulate mRNA levels of *SMN1*/*2.* Nine of the fifteen downregulated genes by C2A–2B–3–4 we validated by qPCR code for factors associated with one or more aspects of neuronal function. These genes were *GFRA1*, *SHTN1*, *PDZD8*, *PCDH19*, *BEX3*, *TUBB2B*, *CNTN1*, *NDRG1* and *BASP1*. Remaining six of the downregulated genes by C2A–2B–3–4 we validated by qPCR code for cancer-associated factors. These genes were *MKI67*, *SAMD5*, *OR51E*, *CD44*, *ERP44* and *POLK*. A recent report showed substantial upregulation of *SMN1* transcripts and SMN protein in LSCC^34^. It remains to be seen if an increase in *SMN1* transcripts in LSCC is also accompanied with the elevated expression of C2A–2B–3–4.

Various mechanisms may account for the regulation of genes by C2A–2B–3–4. Previous finding that C2A–2B–3–4 is prominently localized in the cytosol may suggest that the effect of C2A–2B–3–4 on transcriptome is exerted through regulation of translation of transcription factors^44^. This could be achieved through sponging of specific miRNAs and/or sequestration factors associated with translation. It is also likely that a small fraction of C2A–2B–3–4 remain in the nucleus and modulate transcription of specific genes by interacting directly or indirectly with pol II. SMN is an RNA binding protein with preference for structured RNAs^56^. SMN is directly involved in translation of specific mRNAs in neuronal cells^57^. SMN also regulates transcription through resolution of R-loops^41,58^. Future studies will reveal if regulation of transcription and translation by SMN is mediated by a C2A–2B–3–4/SMN complex.

We performed label-free proteomics to identify proteins with significantly altered expression in cells overexpressing C2A–2B–3–4 and L2A–2B–3–4. We identified 61 upregulated and 57 downregulated proteins that were affected by C2A–2B–3–4. The majority of the affected proteins were specific to C2A–2B–3–4 (Fig. 6). We observed significant consensus between the target proteins affected in our proteomics dataset and genes with altered expression in our RNA-Seq data (Supplementary Table S5). Of the 61 upregulated proteins, expression of the associated transcript was significantly increased in 30 cases, while of the 57 downregulated proteins, the transcript was decreased in 25 cases. In contrast, 12 genes were regulated in the opposite direction at the transcript and protein levels (Supplementary Table S5). In terms of individual validated candidates, *CNDP2* was strongly upregulated in our RNA-Seq and proteomics data, as well as in qPCR and western blot validation (Supplementary Table S5). *NAMPT* was weakly upregulated in RNA-Seq and qPCR, but the protein was strongly affected in both proteomics and western blot. Consistently, there was significant overlap in the affected pathways and GO terms between proteome and RNA-Seq analysis. In particular, both data sets suggested an upregulation of ribosomal components and genes/proteins involved in translation, and downregulation of proteins involved in the cytoskeleton and establishment of cell–cell interactions. CNDP2 was the most upregulated protein captured in our validation by western blot. *CNDP2* gene is located on chromosome 18, not in the enriched chromosomal region (Fig. 2J,K). *CNDP2* codes for a dipeptidase that controls turnover of carnosine and other dipeptides. Dysregulation of CNDP2 has been indicated in colon cancers, PD, male infertility and obesity^59–62^. CNDP2 has been implicated in protection of cells against stress-associated conditions^63^. With significance to neurodegeneration, an early proteomic study found increased levels of CNDP2 in substantia nigra of PD patients^59^. NAMPT, a regulator of NAD metabolism, was the next most upregulated protein we validated by western blot. Loss of NAMPT has been directly linked to impairment of synaptic vesicles endocytosis/exocytosis at the neuromuscular junction (NMJ) as well as degeneration of hippocampus in mice^64,65^. Considering NMJ formation is impaired in SMA, upregulation of NAMPT by C2A–2B–3–4 will have a direct implication for SMA therapy. H1-10 was the third upregulated protein that we reliably validated by western blot. Given the important role played by H1-10 in chromatin modification^66^, its upregulation may support a broader role of C2A–2B–3–4 in modulation of gene expression. Although not statistically significant, results of western blot of many proteins, including SMN, supported the trend towards upregulation, consistent with the findings of RNA-Seq and/or proteomic analysis. Further studies will be needed to discern the combined effects of small changes in protein levels due to overexpression of C2A–2B–3–4.

The role of circRNA in gene regulation is an area of growing significance. However, developing efficient tools to uncover functions of circRNAs remain a challenging task. In general, circRNAs and linear transcripts (mRNAs) are produced from the same precursor transcript. Hence, depletion of a circRNA without affecting its linear counterpart remains an arduous endeavor. Production of circRNAs by transient expressions require efficient transfection and does not guarantee sustained expression of circRNAs. In contrast, production of circRNAs using inducible cell lines stably expressing circRNAs provides a better alternative for overexpression studies, although approach is time consuming and limited to specific cell lines. For the obvious advantages, we chose inducible HEK293 cells stably expressing C2A–2B–3–4. The effect of overexpression of C2A–2B–3–4 revealed by RNA-Seq and proteome analysis bring a unique perspective towards our understanding of *SMN1*/*2* gene function. Many of the functions of C2A–2B–3–4 uncovered here, including transcription, translation and NMJ formation, are dysregulated in SMA. Future studies will reveal if some of functions of C2A–2B–3–4 are shared by other circRNAs such as abundantly expressed C2B-3–4 and as C3-4 generated by *SMN1*/*2*^38^. Carefully designed complementary experiments, including tissue-specific expression and depletion of C2A–2B–3–4 would add to a better understanding of the non-coding functions of *SMN1*/*2*. With direct consequence to the generation of C2A–2B–3–4, a recent report revealed skipping of exon 3 of *SMN1*/*2* upon overexpression of SMN or SMNΔ7^67^. Future experiments would reveal how C2A–2B–3–4 levels would be impacted in pathological conditions, such as LSCC in which SMN is overexpressed^34^. While circRNAs of *SMN1*/*2* were identified ~ 5 years ago, this is the first report on the functions of a *SMN1*/*2* circRNA. We hope our report serves as a catalyst to invite more investigations into the role of *SMN1*/*2* circRNAs in processes associated with SMA and other pathological conditions, including cancer and male infertility.

## Materials and methods

### Mammalian cell culture

Abbreviations used in this publication are listed in Supplementary Table S1. HEK293-derived stable cell lines TC4-2A and TL4-2A were described previously^44^. TC4-2A, TL4-2A and T-REx cells were cultured in Dulbecco’s modified Eagle’s medium (DMEM, Gibco, Cat No. 11965-092), supplemented with 10% fetal bovine serum (Gibco, Cat No. 16000-044) and 1× Glutamax (Gibco, Cat No. 35050061). Antibiotics were added following the manufacturers’ instruction and/or experimentally determined values, including 100 µg/mL zeocin, 15 µg/mL blasiticidin, 100 µg/mL hygromycin (Life technologies, Cat No. R250-01, R210-01, R220-05) and 0.1 µg/mL doxycycline (Dox, Sigma-Aldrich, Cat No. D9891-10G). Cells were cultured in CO_2_ incubator (Thermo Fisher Scientific, NAPCO Series 8000WJ) at 37 °C and 5% CO_2_. For induction of expression, TC4-2A, TL4-2A and control T-REx cells were seeded at a density of 2 × 10^5^ cells per 100 mm dish. After incubating for 18 hours (h), the cell culture medium was replaced with fresh medium containing Dox. After 48 h of incubation, the cell culture medium was replaced with fresh medium containing Dox. The cells were collected 48 h after the second addition of Dox (96 h of induction in total).

### Evaluation of cell doubling time

Cell doubling time was assessed using a method adapted from Dobek et al.^68^ Cells were seeded at a density of 1.5 × 10^4^ per well of 24-well plate in triplicate, with or without Dox. Cells were collected and counted at 6, 12, 24, 36, 48, and 72 h after seeding. Trypan blue solution (Bio-Rad, Cat No. 1450021) was used to count viable cells. Growth curve was plotted to determine the exponential phase. Doubling time was calculated using cell counts from 24 to 72 h, when linear growth was observed.

### RNA isolation and RNase R treatment

Total RNA was isolated from cells using TRIzol reagent (Invitrogen, Cat No. 15596018) following the manufacturer’s instructions. RNA was digested with RQ1 RNase-free DNase (Promega, Cat No. M6101) to remove contaminating genomic DNA, followed by phenol:chloroform extraction and ethanol precipitation. For samples used to amplify circRNA, RNA was treated with RNase R (Applied Biological Material Inc., Cat No. E049), a 3′-to-5′ exoribonuclease to eliminate linear transcripts. RNase R treatment was performed in 10 µL reaction, in which 0.5 µL (10 U) of RNase R was used to treat 2 µg of RNA. The treatment was performed for 45 min (min) at 37 °C, followed by inactivation at 65 °C for 20 min. To estimate the digestion efficiency, mock reactions without enzyme were carried out side by side.

### Reverse transcription and PCR (RT-PCR)

Superscript III reverse transcriptase (RT) (Invitrogen, Cat No. 18080044) was used in cDNA synthesis following the manufacturer’s instruction. Per 5 µL RT reaction, 0.5 µg of total RNA was used. Reactions were primed with either a gene-specific or a random primer (Promega, Cat No. C1181) (Supplementary Table S2). For semi-quantitative PCR, 1 µL of cDNA was added per 20 µL reaction. PCR products were separated by electrophoresis on 6% native polyacrylamide gels and visualized by ethidium bromide staining. For qPCR, 3 µL diluted cDNA (1:20 dilution, equivalent to 15 ng of RNA) was used in a 20 µL qPCR reaction containing 1 × PowerUp SYBR green master mix (Life Technologies, Cat No. A25742) and the desired primer set. Biological triplicates of qPCR reactions were performed on a QuantStudio 3 thermocycler (Thermo Fisher Scientific). Relative expression was determined using the 2^−∆∆Ct^ method using *GAPDH* as housekeeping gene. To determine the copy number of circRNA per cell, we used a standard curve defined by serial dilution of a known quantity of linearized plasmid containing the sequence of the expected PCR product^44^. Of note, the copy number was an average estimated by assuming that the reverse transcription reaction and PCR were performed at 100% efficacy. All primers were obtained from Integrated DNA Technologies. Primers used in this publication are listed in Supplementary Table S2.

### Library generation and RNA-Seq

To confirm RNA integrity, TRIzol-isolated total RNA was characterized using an Agilent Bioanalyzer on an RNA nano chip (RIN ≥ 8). 1 µg of total RNA was then subjected to rRNA depletion using the NEBNext rRNA depletion kit v2 (Human/Mouse/Rat). Libraries were generated from rRNA-depleted RNA using the NEBNext Ultra II directional RNA library prep kit for Illumina. Libraries were barcoded for multiplexing using NEBNext Dual Index oligos for Illumina. Size distribution of libraries was determined using an Agilent Bioanalyzer DNA 1000 chip and concentration was quantified using a Qubit fluorimeter. Libraries were pooled together and sequenced on an Illumina Novaseq 6000 using an S23 flow cell following a 100-cycle, paired-end protocol. Reads from RNA-Seq were mapped to the human reference genome build GRCh38 using HISAT2^69^. For differential expression, mapped reads were assigned to genes according to the Gencode v33 human transcriptome annotation^70^ using the featureCounts script from the Subread software package^71^. Differential expression was estimated using the DESeq2 R package^72^. Raw RNA-Seq data is available from the NCBI sequence read archive under project number PRJNA1058619 and processed data files are available from the Gene Expression Omnibus under accession number GSE255511.

### Protein lysate preparation

Cells were washed in ice-cold phosphate buffered saline (PBS) three times and collected by scraping in 1 mL PBS followed by centrifugation at × 2500*g* for 2 min. PBS supernatants were aspirated, and pelleted cells were lysed using lysis buffer containing 500 mM triethylammonium bicarbonate (TeABC, Sigma–Aldrich, Cat No. 18597-100ML), 1% sodium deoxycholate (SDC, Sigma–Aldrich, Cat No. D5670-5G), and 1 × Halt™ Protease, Phosphatase Inhibitor Cocktail (Thermo Fisher Scientific, 100X, Cat No. 78440)^73^. The lysates were incubated on ice for 45 min, and then sonicated. Samples were centrifuged at 16,000 × *g* for 30 min at 4 °C. Clear supernatants were collected and transferred to new tubes. The purified protein was snap-frozen on dry ice and stored at − 80 °C. Protein concentrations were measured by Bio-Rad protein assay (Bio-Rad, Cat No. 5000006).

### Label-free proteomic quantification and analysis

About 50 µg of each crude protein samples was submitted to the Protein Facility of the Iowa State University Office of Biotechnology (https://www.protein.iastate.edu/). The samples were processed by a label-free relative quantification approach, using the Minora Feature Detector to detect and quantify isotopic clusters. Specifically, the samples were digested with trypsin/Lys-C. SDC was removed by acid precipitation with 2% (v/v) trifluoroacetic acid (TFA). Then, PRTC standard (Pierce Biotechnology, Cat No. 88320) was spiked in to serve as an internal control. The fragmented peptides were then separated by liquid chromatography with tandem mass spectrometry (LC–MS/MS), using the Q Exactive™ Hybrid Quadrupole-Orbitrap Mass Spectrometer system (Thermo Fisher Scientific). The relative abundance of the protein was quantified using peak-area based quantification of the precursor ions of the top three most abundant peptides from the identified protein. Then the raw data was computed using Proteome Discoverer Software (Thermo Fisher Scientific, Version 2.4). The data was searched against Mascot and/or Sequest HT databases. Common contaminants in LC–MS/MS were excluded for downstream analysis^74,75^. MetaboAnalyst platform (https://www.metaboanalyst.ca/, Version 5.0) was used for statistical and bioinformatics analysis, and generation of plots^76^. Features with more than 50% missing values were removed. Interquartile range (IQR) was used for data filtering. Data was normalized by quantile normalization and log transformation. Enrichment analysis was performed by WEB-based GEne SeT AnaLysis Toolkit (http://www.webgestalt.org/, Version 2019, accessed in 2022 December)^77^. Analysis included Gene Ontology terms (GO terms) for biological processes, cellular components and molecular functions. Pathways including those associated with core protein complex subunits, diseases, phenotypes and the chromosomal locations were defined by Kyoto Encyclopedia of Genes and Genomes (KEGG), Panther, Reactome and Wikipathways. The mass spectrometry proteomics data have been deposited to the ProteomeXchange Consortium with identifier PXD048160.

### Western blot

A total amount of 10–50 µg of protein was loaded per lane of a 10% SDS-PAGE gel. Proteins were transferred from gel to a PVDF membrane using a Transblot Turbo fast transfer system (Bio-Rad) after electrophoresis. After transfer and prior to hybridization, membranes were trimmed to remove the region corresponding to the stacking gel. To block the blots, 5% non-fat milk dissolved in Tris-buffered saline containing 0.05% Tween-20 (TBST) was used. Primary antibody incubation was performed at 4 °C overnight with gentle agitation. The dilutions of primary antibodies were prepared as follows: mouse anti-α-tubulin 1:4000 (Sigma-Aldrich, Cat No. T6199), mouse anti-CNDP2 1:500 (Proteintech, Cat No. 14925-1-AP), mouse anti-NAMPT/PBEF (Santa Cruz, sc-166946) 1:500, rabbit anti-H1-10 (also known as H1x, Fortis Life Sciences, Cat No. A304-604A-T) 1:2500. After primary antibody incubation, blots were washed in TBST for 10 min and repeated for a total of three times. Then the blots were incubated with secondary antibodies for 1 h at room temperature, with gentle agitation. The preparation of secondary antibody dilutions was as follows: goat anti-mouse 1:4000 (Jackson ImmunoResearch Laboratories Inc, Cat No. 115-035-003), donkey anti-rabbit 1:2000 (GE Healthcare, Cat No. NA934). After secondary antibody incubation, blots were washed in TBST for 10 min three times and developed using Clarity Western ECL Substrate (Bio-Rad, Cat No. 1705061) or SuperSignal West Femto Maximum Sensitivity Substrate (Thermo Fisher Scientific, Cat No. 34094). The UVP Biospectrum AC imaging system was used to visualize the bands. Quantification of band intensity was performed by using ImageJ software.

### Statistical analysis

Excel (Microsoft, Version 16.62) was used for all the calculation and generation of plots. Data were expressed as mean ± SEM. The unpaired Student’s t-test was applied in statistical analysis. Unless otherwise mentioned, experiments were performed in triplicate, and *p* values were two-tailed and the level of statistical significance was set as *p* < 0.05.

### Supplementary Information

Supplementary Figures.

Supplementary Tables.

## Data Availability

*Accession codes* RNA-Seq data is available from the NCBI sequence read archive under project number PRJNA1058619 and gene expression omnibus under accession number GSE255511. Proteomic data is available from ProteomeXchange Consortium at accession number PXD048160.
