# Supplementary material for: Transcriptome- and proteome-wide effects of a circular RNA encompassing four early exons of the spinal muscular atrophy genes
Source: Sci Rep. 2024 May 7;14:10442. doi: 10.1038/s41598-024-60593-7 (PMC11076517; doi:10.1038/s41598-024-60593-7)
Supplement: Supplementary file 1 — Supplementary Figures. [file 41598_2024_60593_MOESM1_ESM.pdf]

Supplementary Figures for:

Transcriptome- and proteome-wide effects of a circular RNA encompassing four early exons of the spinal muscular atrophy genes

Diou Luo, Eric W. Ottesen, Ji Heon Lee, and Ravindra N. Singh

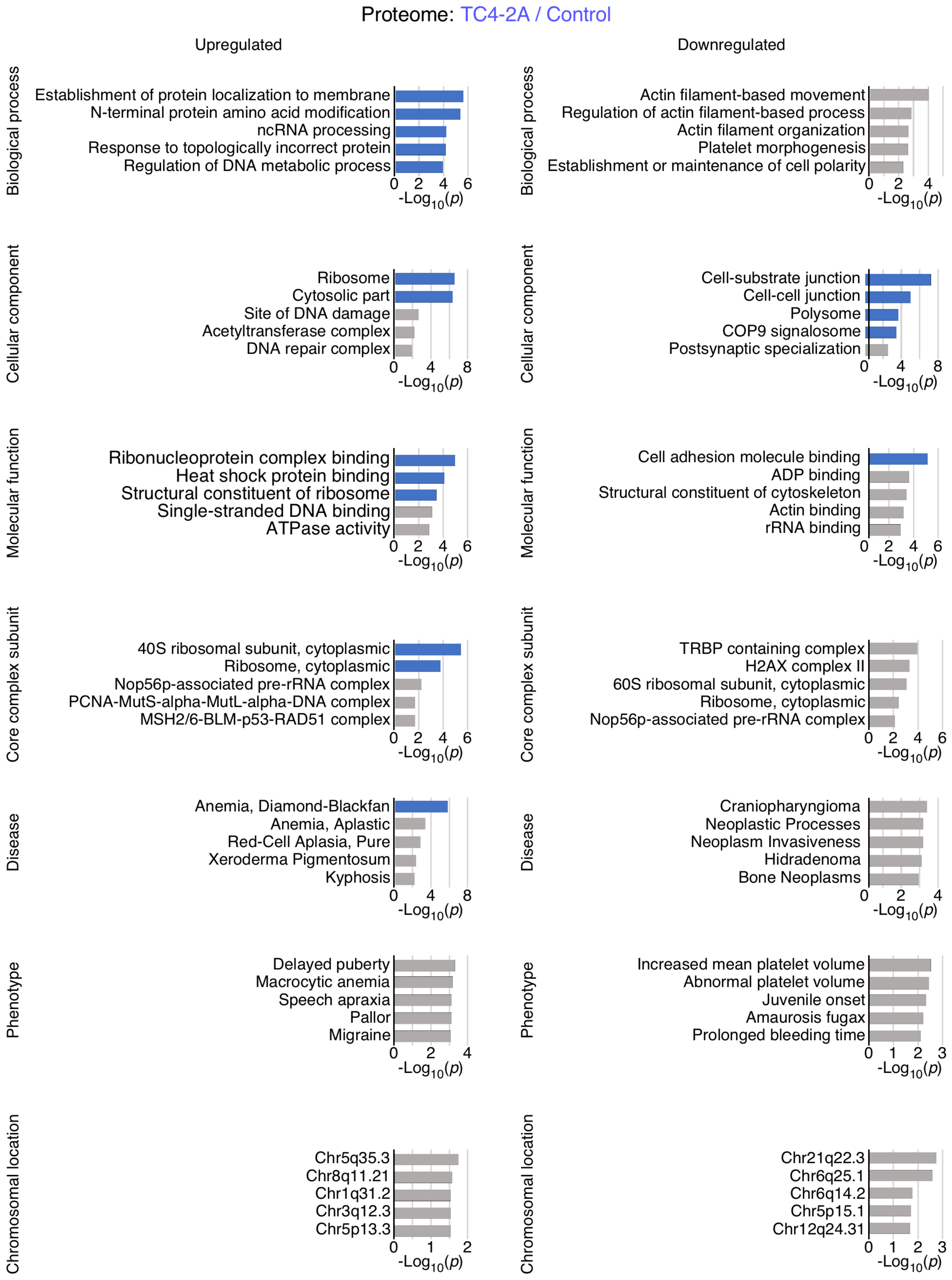

Proteome: TC4-2A / Control
Upregulated
Downregulated
Biological process
Establishment of protein localization to membrane
N-terminal protein amino acid modification
ncRNA processing
Response to topologically incorrect protein
Regulation of DNA metabolic process
Actin filament-based movement
Regulation of actin filament-based process
Actin filament organization
Platelet morphogenesis
Establishment or maintenance of cell polarity
Cellular component
Ribosome
Cytosolic part
Site of DNA damage
Acetyltransferase complex
DNA repair complex
Cell-substrate junction
Cell-cell junction
Polysome
COP9 signalosome
Postsynaptic specialization
Molecular function
Ribonucleoprotein complex binding
Heat shock protein binding
Structural constituent of ribosome
Single-stranded DNA binding
ATPase activity
Cell adhesion molecule binding
ADP binding
Structural constituent of cytoskeleton
Actin binding
rRNA binding
Core complex subunit
40S ribosomal subunit, cytoplasmic
Ribosome, cytoplasmic
Nop56p-associated pre-rRNA complex
PCNA-MutS-alpha-MutL-alpha-DNA complex
MSH2/6-BLM-p53-RAD51 complex
TRBP containing complex
H2AX complex II
60S ribosomal subunit, cytoplasmic
Ribosome, cytoplasmic
Nop56p-associated pre-rRNA complex
Disease
Anemia, Diamond-Blackfan
Anemia, Aplastic
Red-Cell Aplasia, Pure
Xeroderma Pigmentosum
Kyphosis
Craniopharyngioma
Neoplastic Processes
Neoplasm Invasiveness
Hidradenoma
Bone Neoplasms
Phenotype
Delayed puberty
Macrocytic anemia
Speech apraxia
Pallor
Migraine
Increased mean platelet volume
Abnormal platelet volume
Juvenile onset
Amaurosis fugax
Prolonged bleeding time
Chromosomal location
Chr5q35.3
Chr8q11.21
Chr1q31.2
Chr3q12.3
Chr5p13.3
Chr21q22.3
Chr6q25.1
Chr6q14.2
Chr5p15.1
Chr12q24.31
-Log10(p)

Supplementary Figure S1. Over-representation analysis (ORA) of significantly regulated proteins of TC4-2A compared to T-REx control. The top 5 most significant enriched results are shown. Analyses of upregulated genes are shown on the left side and analyses of downregulated genes on the right side. Bars are color-coded to indicate the False Discovery Rate (FDR) adjusted p values: blue, FDR < 0.05; grey, FDR $\geqslant$ 0.5, un-adjusted $p < 0.05$. The bar size shows the $-\log_{10}$ transformed un-adjusted p value.

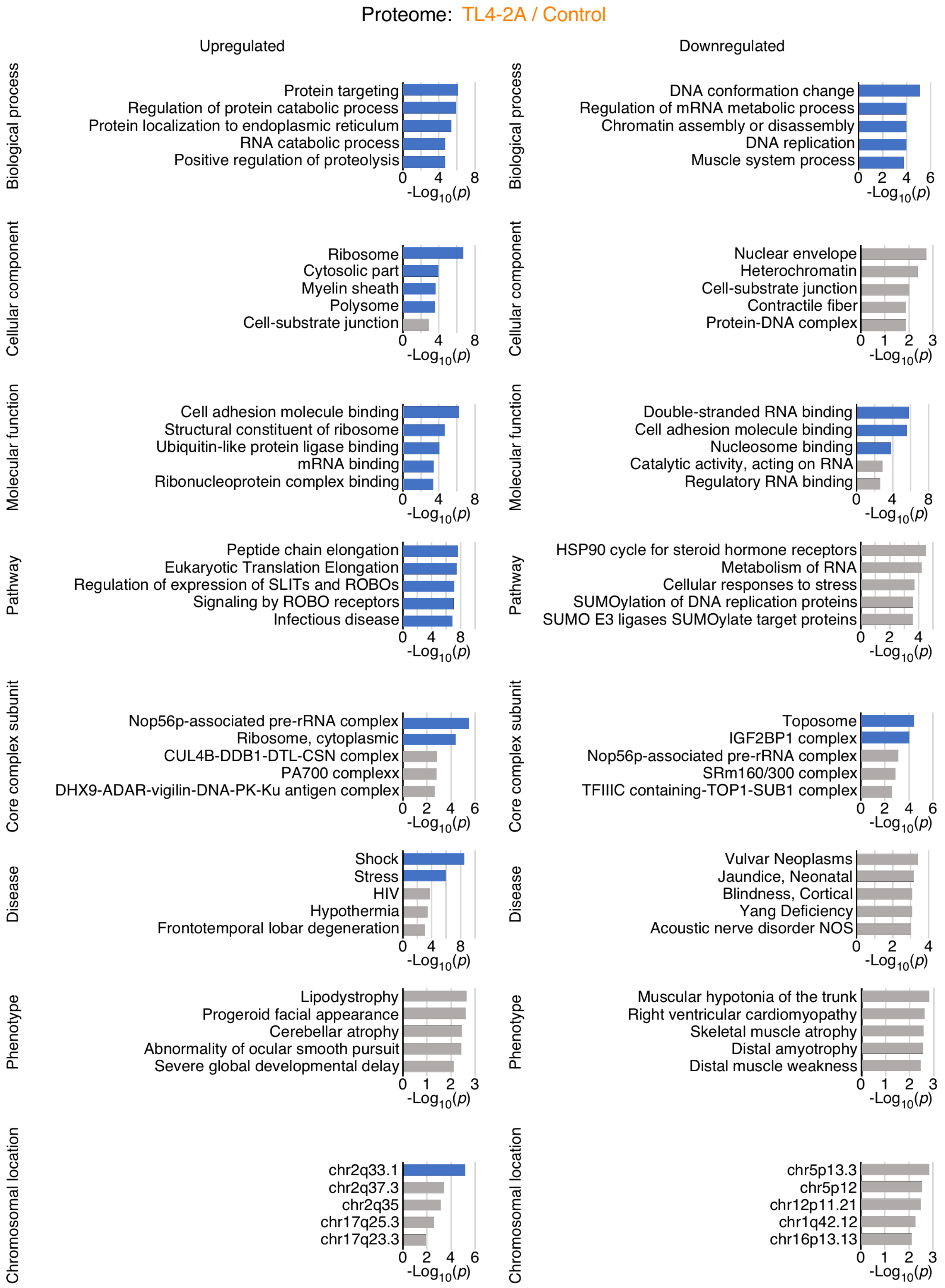

Proteome: TL4-2A / Control
Upregulated
Downregulated
Biological process
Protein targeting
Regulation of protein catabolic process
Protein localization to endoplasmic reticulum
RNA catabolic process
Positive regulation of proteolysis
DNA conformation change
Regulation of mRNA metabolic process
Chromatin assembly or disassembly
DNA replication
Muscle system process
Cellular component
Ribosome
Cytosolic part
Myelin sheath
Polysome
Cell-substrate junction
Nuclear envelope
Heterochromatin
Cell-substrate junction
Contractile fiber
Protein-DNA complex
Molecular function
Cell adhesion molecule binding
Structural constituent of ribosome
Ubiquitin-like protein ligase binding
mRNA binding
Ribonucleoprotein complex binding
Double-stranded RNA binding
Cell adhesion molecule binding
Nucleosome binding
Catalytic activity, acting on RNA
Regulatory RNA binding
Pathway
Peptide chain elongation
Eukaryotic Translation Elongation
Regulation of expression of SLITs and ROBOs
Signaling by ROBO receptors
Infectious disease
HSP90 cycle for steroid hormone receptors
Metabolism of RNA
Cellular responses to stress
SUMOylation of DNA replication proteins
SUMO E3 ligases SUMOylate target proteins
Core complex subunit
Nop56p-associated pre-rRNA complex
Ribosome, cytoplasmic
CUL4B-DDB1-DTL-CSN complex
PA700 complexx
DHX9-ADAR-vigilin-DNA-PK-Ku antigen complex
Toposome
IGF2BP1 complex
Nop56p-associated pre-rRNA complex
SRm160/300 complex
TFIIIC containing-TOP1-SUB1 complex
Disease
Shock
Stress
HIV
Hypothermia
Frontotemporal lobar degeneration
Vulvar Neoplasms
Jaundice, Neonatal
Blindness, Cortical
Yang Deficiency
Acoustic nerve disorder NOS
Phenotype
Lipodystrophy
Progeroid facial appearance
Cerebellar atrophy
Abnormality of ocular smooth pursuit
Severe global developmental delay
Muscular hypotonia of the trunk
Right ventricular cardiomyopathy
Skeletal muscle atrophy
Distal amyotrophy
Distal muscle weakness
Chromosomal location
chr2q33.1
chr2q37.3
chr2q35
chr17q25.3
chr17q23.3
chr5p13.3
chr5p12
chr12p11.21
chr1q42.12
chr16p13.13
-Log10(p)

Supplementary Figure S2. Over-representation analysis (ORA) of significantly regulated proteins of TL4-2A compared to T-REx control. The top 5 most significant enriched results ($p<0.05$) are shown. Analyses of upregulated genes are shown on the left side and analyses of downregulated genes on the right side. Bars are color-coded to indicate the False Discovery Rate (FDR) adjusted p values: blue, FDR < 0.05; grey, FDR $\geqslant$ 0.5, un-adjusted $p < 0.05$.

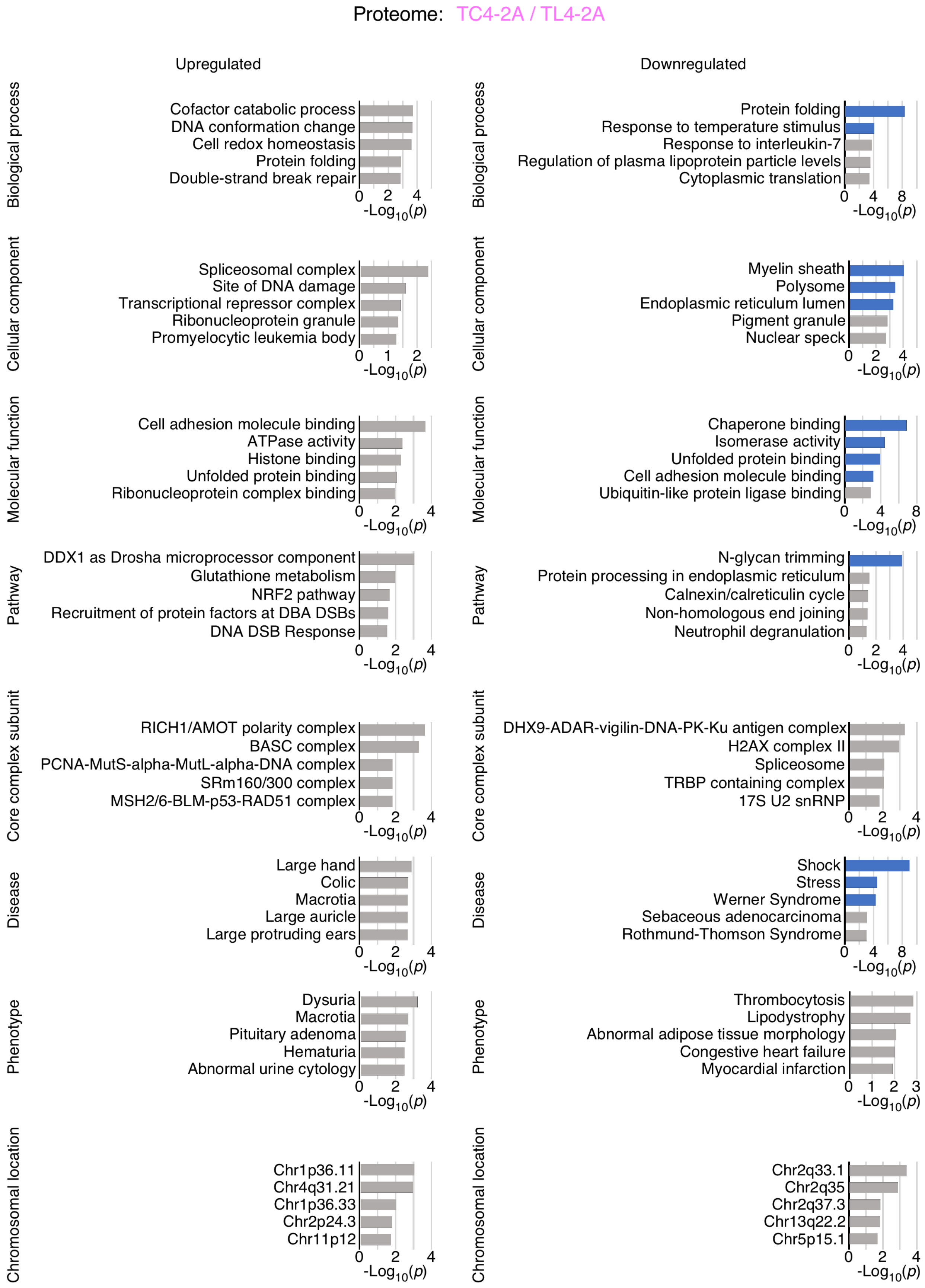
Proteome: TC4-2A / TL4-2A
Upregulated
Downregulated
Biological process
Cofactor catabolic process
DNA conformation change
Cell redox homeostasis
Protein folding
Double-strand break repair
Protein folding
Response to temperature stimulus
Response to interleukin-7
Regulation of plasma lipoprotein particle levels
Cytoplasmic translation
Cellular component
Spliceosomal complex
Site of DNA damage
Transcriptional repressor complex
Ribonucleoprotein granule
Promyelocytic leukemia body
Myelin sheath
Polysome
Endoplasmic reticulum lumen
Pigment granule
Nuclear speck
Molecular function
Cell adhesion molecule binding
ATPase activity
Histone binding
Unfolded protein binding
Ribonucleoprotein complex binding
Chaperone binding
Isomerase activity
Unfolded protein binding
Cell adhesion molecule binding
Ubiquitin-like protein ligase binding
Pathway
DDX1 as Drosha microprocessor component
Glutathione metabolism
NRF2 pathway
Recruitment of protein factors at DBA DSBs
DNA DSB Response
N-glycan trimming
Protein processing in endoplasmic reticulum
Calnexin/calreticulin cycle
Non-homologous end joining
Neutrophil degranulation
Core complex subunit
RICH1/AMOT polarity complex
BASC complex
PCNA-MutS-alpha-MutL-alpha-DNA complex
SRm160/300 complex
MSH2/6-BLM-p53-RAD51 complex
DHX9-ADAR-vigilin-DNA-PK-Ku antigen complex
H2AX complex II
Spliceosome
TRBP containing complex
17S U2 snRNP
Disease
Large hand
Colic
Macrotia
Large auricle
Large protruding ears
Shock
Stress
Werner Syndrome
Sebaceous adenocarcinoma
Rothmund-Thomson Syndrome
Phenotype
Dysuria
Macrotia
Pituitary adenoma
Hematuria
Abnormal urine cytology
Thrombocytosis
Lipodystrophy
Abnormal adipose tissue morphology
Congestive heart failure
Myocardial infarction
Chromosomal location
Chr1p36.11
Chr4q31.21
Chr1p36.33
Chr2p24.3
Chr11p12
Chr2q33.1
Chr2q35
Chr2q37.3
Chr13q22.2
Chr5p15.1
$-Log_{10}(p)$

Supplementary Figure S3. Over-representation analysis (ORA) of significantly regulated proteins of TC4-2A compared to TL4-2A control. The top 5 most significant enriched results ($p<0.05$) are shown. Analyses of upregulated genes are shown on the left side and analyses of downregulated genes on the right side. Bars are color-coded to indicate the False Discovery Rate (FDR) adjusted p values: blue, FDR < 0.05; grey, FDR $\geqslant 0.5$, un-adjusted $p < 0.05$. The bar size shows the $-\log_{10}$ transformed un-adjusted p value.

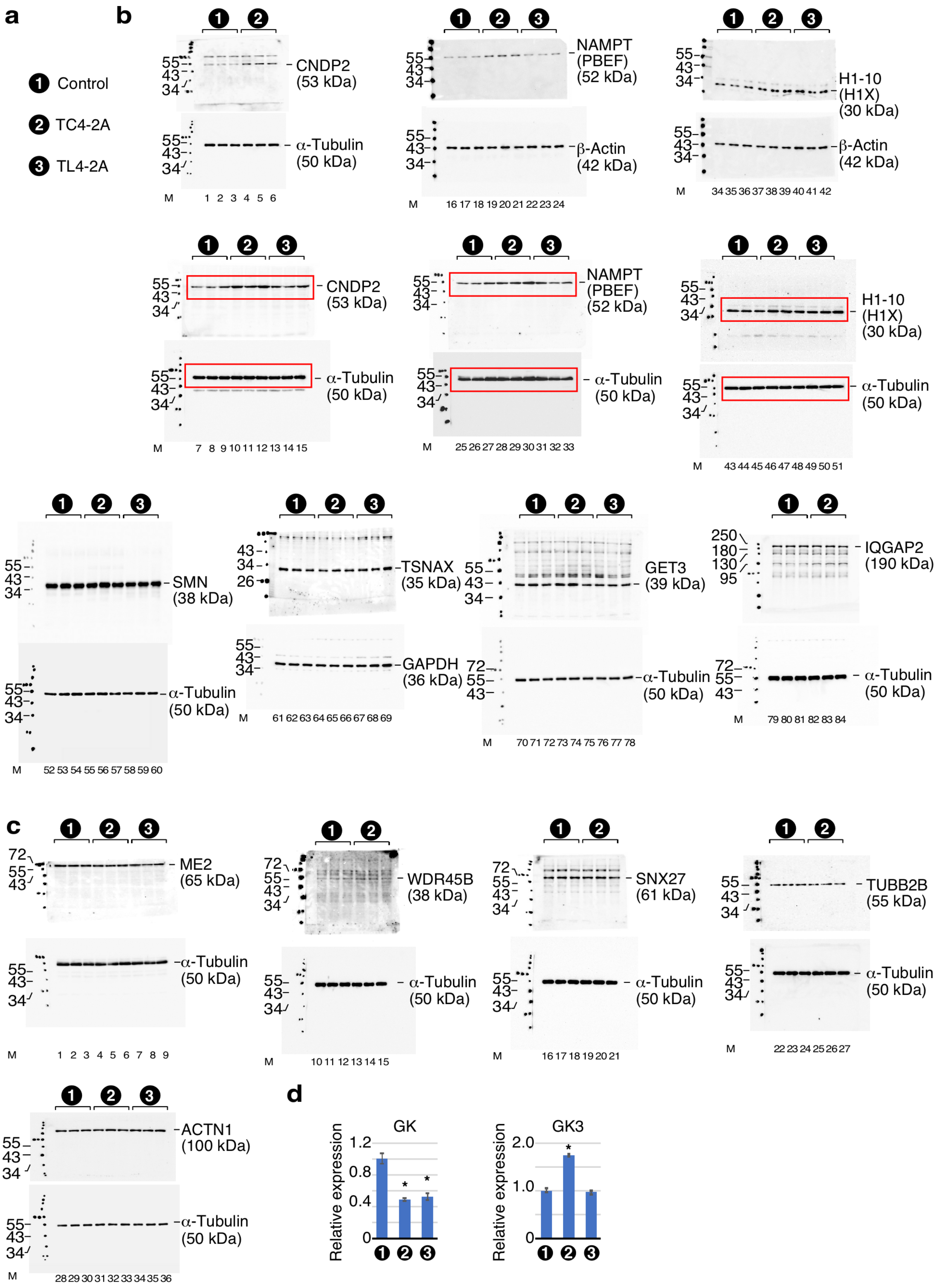
a
b
c
d
1 Control
2 TC4-2A
3 TL4-2A
CNDP2 (53 kDa)
α-Tubulin (50 kDa)
NAMPT (PBEF) (52 kDa)
β-Actin (42 kDa)
H1-10 (H1X) (30 kDa)
SMN (38 kDa)
TSNAX (35 kDa)
GAPDH (36 kDa)
GET3 (39 kDa)
IQGAP2 (190 kDa)
ME2 (65 kDa)
WDR45B (38 kDa)
SNX27 (61 kDa)
TUBB2B (55 kDa)
ACTN1 (100 kDa)
GK
GK3
Relative expression
M

Supplementary Figure S4. Attempts to validate representative candidates identified in proteomics analysis. (A) Numbering of cell lines, consistent throughout the figure. **(B)** Western blot results of upregulated candidate proteins. For the top three upregulated candidates two independent blots are shown. Regions of blots used for representative images in Figure 7 are indicated with red boxes. α-tubulin, β-actin and GAPDH are used as loading controls. Cell type is indicated at the top of the panel. Antibody used is indicated at the right and nearby molecular weight markers are indicated at the left. **(C)** Western blot results of downregulated candidates. Labeling is the same as in (B). **(D)** qPCR quantification of mRNA expression levels of *GK* and its pseudogene *GK3 (GK3P)*. Cell lines are indicated under the X axis. The Y axis represents the relative expression as compared with control, T-REx cells. Error bars represent standard error of the mean. Statistical significance: n = 3 *, p<0.05.
